# *MicroRNA29a* regulates IL-33-mediated tissue remodelling in tendon disease

**DOI:** 10.1038/ncomms7774

**Published:** 2015-04-10

**Authors:** Neal L. Millar, Derek S. Gilchrist, Moeed Akbar, James H. Reilly, Shauna C. Kerr, Abigail L. Campbell, George A. C. Murrell, Foo Y. Liew, Mariola Kurowska-Stolarska, Iain B. McInnes

**Affiliations:** 1 Institute of Infection, Immunity and Inflammation, College of Medicine, Veterinary and Life Sciences University of Glasgow, Glasgow G12 8QQ, UK; 2 Orthopaedic Research Institute, Department of Orthopaedic Surgery, St George Hospital Campus, University of New South Wales, Sydney, New South Wales NSW 2217, Australia; 3 School of Biology and Basic Medical Sciences, Soochow University, Suzhou 215006, China

## Abstract

MicroRNA (miRNA) has the potential for cross-regulation and functional integration of discrete biological processes during complex physiological events. Utilizing the common human condition tendinopathy as a model system to explore the cross-regulation of immediate inflammation and matrix synthesis by miRNA we observed that elevated IL-33 expression is a characteristic of early tendinopathy. Using *in vitro* tenocyte cultures and *in vivo* models of tendon damage, we demonstrate that such IL-33 expression plays a pivotal role in the transition from type 1 to type 3 collagen (Col3) synthesis and thus early tendon remodelling. Both IL-33 effector function, via its decoy receptor sST2, and Col3 synthesis are regulated by *miRNA29a*. Downregulation of *miRNA29a* in human tenocytes is sufficient to induce an increase in Col3 expression. These data provide a molecular mechanism of miRNA-mediated integration of the early pathophysiologic events that facilitate tissue remodelling in human tendon after injury.

## Abstract (web summary)

Collagen 3 is increased during tendon repair, but is then replaced by Collagen 1 that has superior biomechanical properties. Here the authors show that IL-33 is induced by tendon damage and regulates miR-29a, which controls Collagen 3 production and feeds back on IL-33, orchestrating tendon repair.

## Graphical abstract

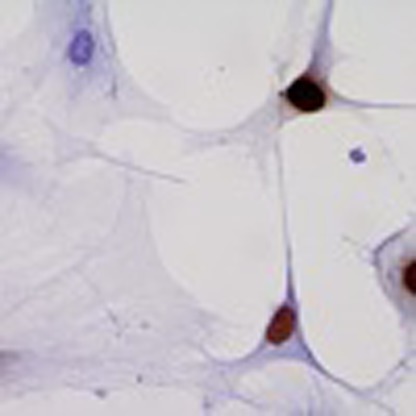

Dysregulated tissue repair and inflammation characterize many common musculoskeletal pathologies^1^, including tendon disorders. Tendinopathies represent a common precipitant for musculoskeletal consultation in primary care^2^,^3^ and comprise 30–50% of all sports injuries^3^. Tendinopathy is characterized by altered collagen production from subtype 1 to 3 resulting in a decrease in tensile strength that can presage clinical tendon rupture^4^. Inflammatory mediators are considered crucial to the onset and perpetuation of tendinopathy^5^. Expression of various cytokines has been demonstrated in inflammatory cell lineages and tenocytes suggesting that both infiltrating and resident populations participate in pathology^6^,^7^,^8^,^9^. Mechanical properties of healing tendons in interleukin (IL)-6-deficient mice are inferior compared with normal controls^10^, while tumour necrosis factor-α blockade improves the strength of tendon–bone healing in a rat tendon injury model^11^. While these data raise the intriguing possibility that cytokine targeting could offer therapeutic utility, there is currently insufficient mechanistic understanding of cytokine/matrix biology in tendon diseases to manifest this possibility in practice.

IL-33 is a member of the IL-1 cytokine family that plays a major role in innate and acquired immune responses. IL-33 is expressed in endothelial cells and fibroblasts, co-located with chromatin in the nucleus^12^. IL-33 is released following cellular damage^13^ and biomechanical overload^14^, and is thus considered an ‘alarmin'^15^. It has been implicated in a variety of inflammatory pathologies including pulmonary, cutaneous and articular diseases^16^. IL-33 functions via its cognate receptor ST2 that exists in membrane-bound (mST2) or soluble decoy form (sST2) and signals via a canonical IL-1R signalling cascade. Cytokines are often regulated at the post-transcriptional level by miRNA that control the gene expression by translational suppression and destabilization of target mRNAs^17^. miRNA networks are emerging as key homeostatic regulators of tissue repair with fundamental roles proposed in stem cell biology, inflammation, hypoxia response and angiogenesis^18^.

On the basis of the emerging role of fibroblast-derived IL-33 in inflammatory^19^,^20^ and fibrotic disorders^21^ and previous investigations showing increased inflammatory leukocyte infiltration in torn rotator cuff tendons^22^, we hypothesized that the IL-33/ST2 signalling pathway might play a significant role in tendon pathology. Our data derived in animal and human models of tendinopathy collectively suggest that the IL-33/ST2 system functions as an alarmin in tendon by triggering inflammation and switching collagen production towards biomechanically inferior collagen III synthesis, thus contributing to the severity of tendinopathy. Importantly, we found that *miR-29a* acts as a critical regulator of tenocyte biology by integrating IL-33 effector function and collagen matrix changes. This provides a novel insight into the coordinated regulation of disparate biochemical pathways by an miRNA to thereby modulate emerging tissue pathology.

## Results

### IL-33 and ST2 expression in human tendinopathy

**Figure 1:**
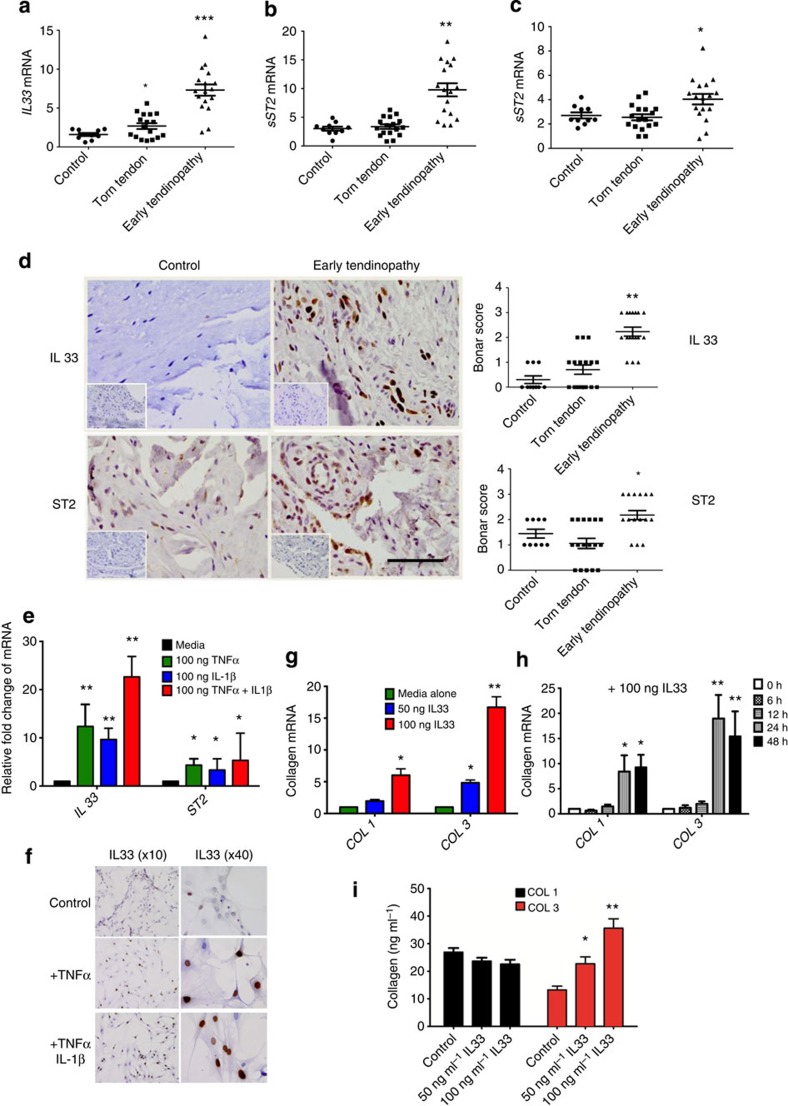
IL-33/ST2 expression in human tendon. (**a**) *IL-33*, (**b**) soluble ST2 (*sST2*) and (**c**) *mST2* mRNA expression in human tendon samples. Fold change in gene expression of IL-33, sST2 and mST2 in control (semi-membranosus tendon, *n*=10), torn supraspinatus tendon (established pathology) and matched subscapularis human tendon samples (early pathology; *n*=17). Data are mean±s.d. relative to the housekeeping gene18S (mean of duplicate analysis). *P<0.05, **P<0.01, ***P<0.001 versus control (Student's *t-*test). (**d**) Immunostaining of IL-33 and ST2 in control (*n*=10), torn tendon (*n*=17) and early tendinopathy (*n*=17). Graphs illustrate modified Bonar scoring based on 10 high-power fields. Data are mean±s.d. *P<0.05, **P<0.01 versus control (Student's *t*-test). Scale bar, 65 μm. (**e**) Fold change in gene expression of *IL33* and *ST2* 24 h post incubation with tumour necrosis factor (TNF-α), IL-1β or in combination depicting relative expression to media alone utilizing housekeeping gene *GAPDH*. Data are mean±s.d. of triplicate samples, representative of three individual patient samples. *P<0.05, **P<0.01 versus control (media) (Student's *t*-test). (**f**) IL-33 immunostaining of human tendon explants cultured for 24 h with medium (control), 100 ng ml^−1^ TNFα or 100 ng ml^−1^ TNFα+100 ng ml^−1^ IL-1β. (**g**) Fold change in *COL1* and *COL3* mRNA expression in human tendon explants cultured for 24 h with rhIL-33, relative to housekeeping gene *GAPDH*. (**h**) Time course of *COL1* and *COL3* mRNA expression following incubation with rhIL-33, relative to housekeeping gene *GAPDH*. (**i**) Collagen 1 and 3 protein expression in human tendon explants 24 h post incubation with rhIL-33. For (**g**–**i**) data are mean±s.d. of triplicate samples, representative of three individual patients. *P<0.05, **P<0.01 versus control (Student's *t*-test).

We first investigated IL-33 expression in human tendinopathy using our previously developed model^23^. *IL33*, soluble and membrane-bound *ST2* transcripts were significantly upregulated in early tendinopathy compared with control or torn tendon biopsies (Fig. 1a–c). Early tendinopathy tissues exhibited significantly greater staining for IL-33 and ST2 compared with torn tendon or control biopsies (Fig. 1d). Staining was prominent in endothelial cells (CD34^+^) and particularly fibroblast-like cells, namely tenocytes, which are considered pivotal to the regulation of early tendinopathy (Supplementary Fig. 1a,b). In parallel, *in vitro*-cultured tenocytes expressed nuclear IL-33 that was upregulated at both the mRNA and protein levels following stimulation by TNF-α and IL-1β (Fig. 1e,f). In contrast, ST2 was constitutively expressed in both resting and unstimulated tenocytes (Supplementary Fig. 1c).

### IL-33 regulates tenocyte collagen matrix and cytokine synthesis

Matrix dysregulation towards collagen 3 (Col3) expression is a key early phenotypic change in tendinopathy thereby hastening repair; Col3 is however biomechanically inferior^4^. IL-33 induced dose- and time-dependent upregulation of total collagen protein (Supplementary Fig. 1d), accounted for by an increased expression of type I but particularly type III collagen mRNA and protein (Fig. 1g–i, Supplementary Fig. 1e). Following array analysis (Supplementary Fig. 2a–c) and consistent with reported IL-33 downstream signalling^12^,^16^. IL-33-induced collagen expression was abrogated by ERK and NFκB inhibition (Supplementary Fig. 2d,e). Recombinant IL-33 also significantly elevated the production of IL-6, IL-8 and monocyte chemoattractant protein-1, which was abrogated by NF-κB inhibition (Supplementary Fig. 2f), suggesting that IL-33 operates in tenocytes via its canonical IL-1R signalling pathway.

### Modelling IL-33/ST2 pathway *in vivo* following tendon injury

**Figure 2:**
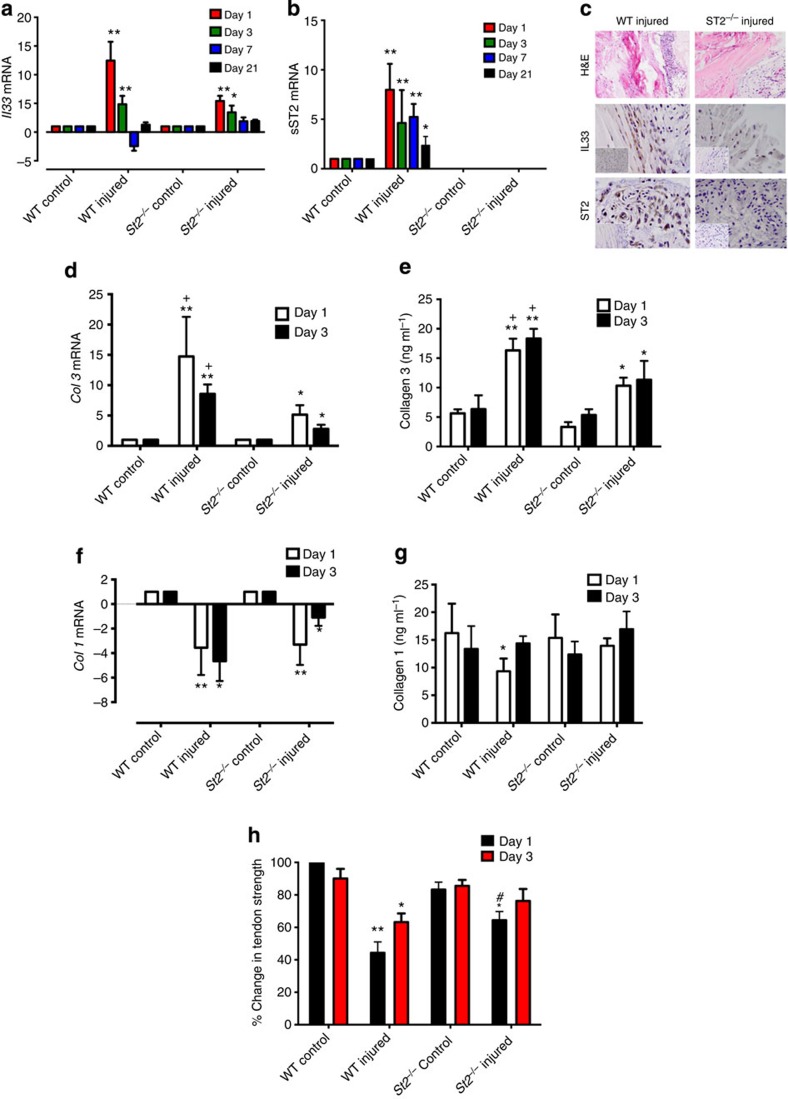
IL-33/ST2 axis in tendon healing *in vivo*. Kinetics of (**a**) *Il33* and (**b**) soluble *St2* gene expression post injury. Data are mean fold change±s.d. showing relative expression to *18s* housekeeping gene (pooled data from four mice per group performed on four sequential occasions, *n*=16 per condition). *P<0.05, **P<0.01 versus control (WT) mice (ANOVA) +*P*<0.05,++*P*<0.01, NS not significant; control (WT) injured versus *St2*^*−/−*^ injured. (**c**) Immunohistochemistry showing IL-33 and ST2 expression in tendon biopsies of WT and *St2*^*−/−*^ mice day 1 post injury. IgG control shown in bottom left of pictures. Black horizontal line indicates 50 μm. (**d**) *Col3* mRNA and (**e**) Collagen 3 protein levels in tendon biopsies of WT and *St2*^*−/−*^ mice post injury. (**f**) *Col1* mRNA and (**g**) Collagen 1 protein levels in tendon biopsies of WT and *St2*^*−/−*^ mice post injury. For (**d**–**g**) data are mean±s.d. of duplicate samples, representative of four mice per condition (*n*=16). *P<0.05, **P<0.01 versus control (WT) mice. +*P*<0.05, ++*P*<0.01 control (WT) injured versus *St2*^*−/−*^ injured mice (ANOVA). mRNA graphs show relative expression to *18s* housekeeping gene. (**h**) Percentage change in tendon strength for WT and *St2*^*−/−*^ mice post injury. Data are mean±s.d., representative of four mice per condition (*n*=16). *P<0.05, **P<0.01 versus control mice. ^#^*P*<0.05 *St2*^*−/−*^ injured versus WT injured mice (Mann–Whitney *U*-test).

We extended these observations to a well-established *in vivo* murine model of tendon injury. IL-33 mRNA and protein were elevated on days 1 and 3 post tendon injury in wild-type (WT) BALB/c mice (Fig. 2a–c). This was significantly reduced in injured *St2*^*−/−*^ BALB/c mice suggesting autocrine regulation. Soluble *St2* was significantly upregulated at all time points post injury in WT mice compared with uninjured controls (Fig. 2b). No significant changes in *Il33* or *St2* transcript were found in WT mice at days 7 or 21 post injury, or for *Il33* expression in *St2*^*−/−*^ mice, suggesting that the impact of IL-33 expression is manifest early, in keeping with ‘alarmin' type activity in tendon injury/repair.

Analysis of collagen synthesis revealed significantly greater expression of collagen 3 at all time points post injury in WT mice compared with uninjured controls or injured *St2*^*−/−*^ mice (Fig. 2d,e and Supplementary Fig. 3). Collagen 1 was initially downregulated (days 1, 3) at mRNA levels (Fig. 2f) in injured WT mice but reverted towards pre-injury levels by days 7 and 21 (Supplementary Fig. 3a) with a similar trend in Collagen 1 protein expression (Fig. 2g). In contrast, injured *St2*^*−/−*^ mice showed prolonged reduction of collagen 1 synthesis (days 1, 3 and 7) returning to baseline only by day 21 (Supplementary Fig. 3d). Importantly, injury of WT mice tendons resulted in a significant decrease in biomechanical strength at day 1 post injury compared with that of the *St2*^*−/−*^ mice (Fig. 2h). The biomechanical strength in all injured mice recovered by days 7 and 21 (Supplementary Fig. 3f). These data suggest altered collagen matrix synthesis in *St2*^*−/−*^ mice implicating IL-33/ST2 as an early modulator of collagen changes in tendon injury that has biomechanical significance.

### IL-33 modifies collagen 3 expression *in vivo*

**Figure 3:**
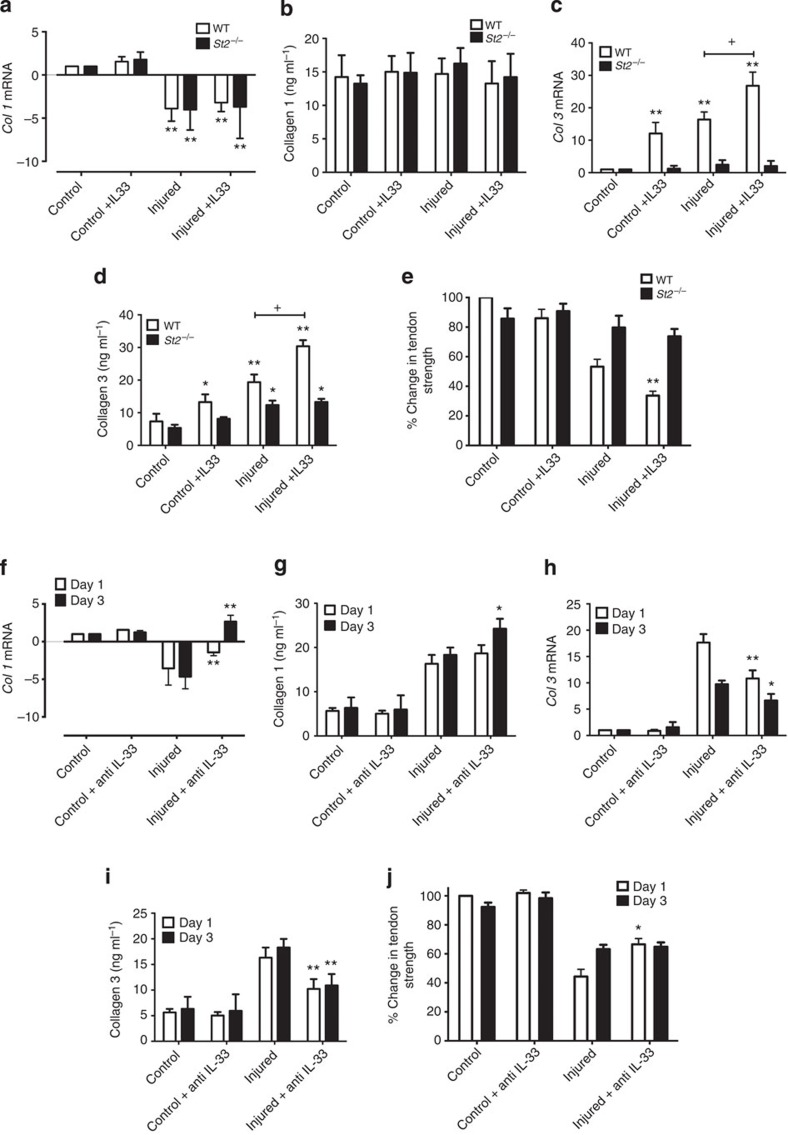
IL-33/anti-IL-33 effects on collagen production and tendon strength *in vivo*. (**a**) *Col1* mRNA, (**b**) Collagen 1 protein, (**c**) *Col3* mRNA and (**d**) Collagen 3 protein in WT and *St2*^*−/−*^ mice on day 1 post injury. mRNA graphs show relative expression to *18s* housekeeping gene. Data are mean±s.d. of duplicate samples, representative of four mice per condition (*n*=16). *P<0.05,**P<0.01, injured versus uninjured mice.^+^*P*<0.05 WT versus ST2^−/−^ mice. (ANOVA). (**e**) Percentage change in tendon strength in WT and St2^−/−^ mice on day 1 post injury with or without rhIL-33 treatment. Data are mean±s.d., representative of four mice per group (*n*=16). **P<0.01, injured versus injured+IL-33 mice (Mann–Whitney *U*-test). (**f**) *Col1* mRNA, (**g**) Collagen 1 protein, (**h**) *Col3* mRNA, (**i**) Collagen 3 protein levels and (**j**) percentage change in tendon strength post tendon injury in WT mice with or without anti-IL-33 treatment. Data for mRNA show relative expression to *18s* housekeeping gene. Data are mean±s.d., representative of four mice per condition (*n*=16). *P<0.05, **P<0.01, injured versus uninjured mice (Mann–Whitney *U*-test).

To confirm that IL-33 regulates collagen synthesis during tendon injury, we sought to directly modify IL-33 effector function *in vivo*. Administration of rhIL-33 did not affect collagen 1 synthesis (Fig. 3a,b), but significantly increased collagen 3 synthesis particularly in injured tendons (Fig. 3c,d and Supplementary Fig. 4a,b). Moreover, rhIL33 administration significantly reduced ultimate tendon strength at all time points post injection in WT mice (Fig. 3e and Supplementary Fig. 4c,d) suggesting that such changes were of functional impact. IL-33 administration did not affect collagen matrix synthesis or ultimate tendon strength of the healing tendon in *St2*^*−/−*^ mice confirming that IL-33 acted via an ST2-dependent pathway (Supplementary Fig. 4).

We next directly targeted IL-33 *in vivo*. Neutralizing antibodies to IL-33 attenuated the collagen 1 to 3 switch at days 1 and 3 post injury in injured WT mice (Fig. 3f–i, Supplementary Fig. 5a) resulting in a significant increase in biomechanical strength at day 1 post injury in the tendons of WT mice (Fig. 3j). This effect was not seen at later time points (Supplementary Fig. 5b). In control experiments, we observed no effect of rIL-33 on *St2*^*−/−*^ mice (Supplementary Fig. 5c,d). These results, therefore, further demonstrate the contribution of endogenous IL-33 to early injury-induced tendinopathy.

### IL33 regulates collagen 3 via *miR-29a* in tenocytes

**Figure 4:**
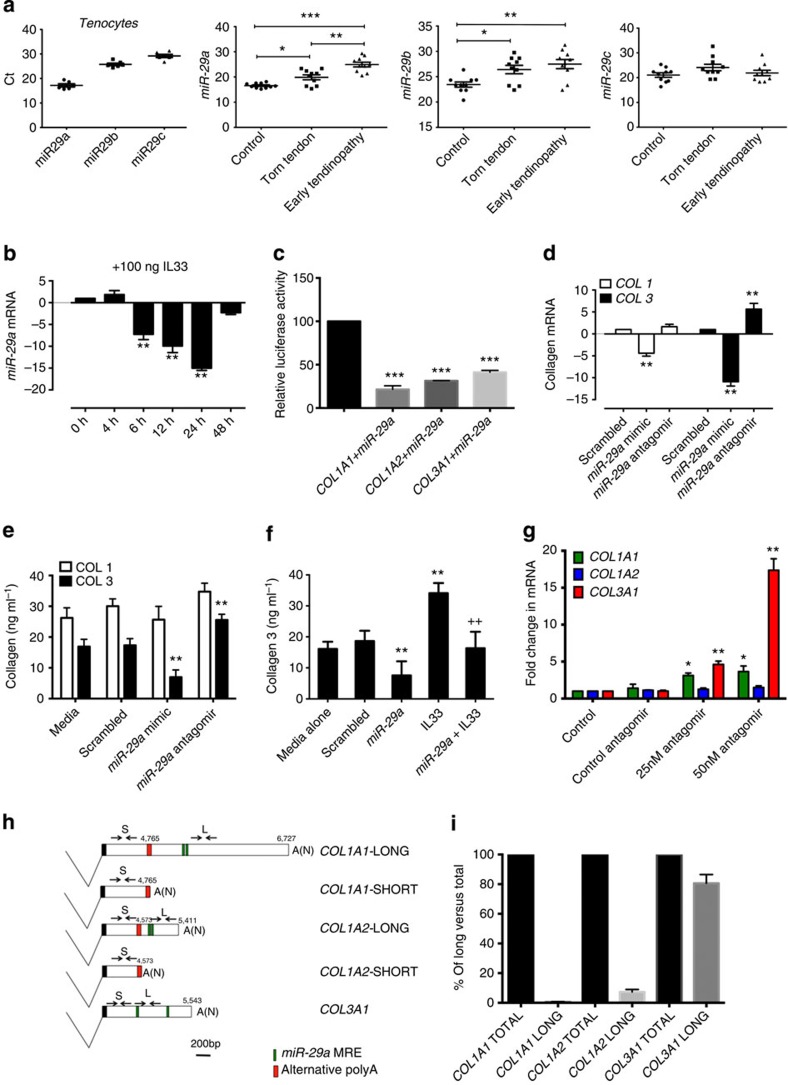
IL33 promotes differential regulation of collagen 1 and 3 via *miR-29a* in human tenocytes. (**a**) Expression (*C*t, threshold cycle values; lower *C*t=more abundance expression) of *miR-29a*, *miR-29b* and *miR-29c* in tenocytes (*n*=6) and relative fold change to *U6* housekeeping in control, torn supraspinatus (torn tendon) and matched subscapularis tendon (early tendinopathy). Data are mean±s.d. of duplicate samples and represent experiments on 10 patient samples. *P<0.05, **P<0.01. (Student's *t*-test). (**b**) Time course of *miR-29a* expression in human tenocytes following cultured with rhIL-33 showing relative fold change to *U6* housekeeping. (**c**) Luciferase activity in primary human tenocytes transfected with precursor *miR-29a* containing 3′UTR of *COL1A1*, *COL1A2* or *COL3A1*. Activity was determined relative to controls transfected with scrambled RNA, which was defined as 100%. This was repeated in three independent experiments. **P<0.01, **P<0.01 versus scrambled control. (Student's *t*-test). (**d**) *COL1* and *COL3* mRNA and (**e**) collagen 1 and 3 protein expression following transfection with scrambled mimic, *miR-29a* mimic or *miR-29a* antagomir. (**f**) Collagen 3 protein levels following addition of *miR-29a* mimic/antagomir and 100 ng rhIL-33. For **c**–**f**, data shown are mean±s.d. of duplicate samples and represent experiments on five tendon explant samples, (*n*=5). *P<0.05, **P<0.01 versus scrambled control, ^++^P<0.01 versus without *miR-29a*. (ANOVA). (**g**) *COL1A1*, *COL1A2* and *COL3A1* mRNA in human tenocytes following transfection with scrambled mimic (control antagomir) or *miR-29a* antagomir showing relative fold change to *U6* housekeeping. Data are mean±s.d. of duplicate samples and represent experiments on three tendon explant samples. (*n*=3). *P<0.05, **P<0.01 versus control (Mann–Whitney *U*-test). (**h**) Schematic diagram of alternative polyadenylated 3′UTRs from *COL1A1*, *COL1A2 and COL3A1*, showing the positions of *miR-29a*-binding sites, qPCR primers and polyadenylation signals. A (N), polyadenylation. (**i**) Relative contribution of the long 3′UTR forms of *COL1A1*, *COL1A2* and *COL3A1* to their total transcript levels was measured by qPCR (location of primers shown in **h**, *n*=3).

Having established that IL-33 drives the differential regulation of collagen 1 and 3 in tenocytes, we postulated a mechanistic role for the miRNA network in this process. Previous studies have shown that the *miR-29* family directly targets numerous extracellular matrix genes, including type 1 and 3 collagens^24^,^25^ and is implicated in the regulation of innate and adaptive immunity^26^. Computational algorithms predict that *miR-29* may also target *sST2* (ref. 27). We found that all members of the *miR-29* family were expressed in human tendon biopsies and explanted tenocytes (Fig. 4a) with *miR-29a* showing the most altered expression in early tendinopathy biopsies. In tenocyte culture, IL-33 significantly reduced the expression of *miR-29a* at 6, 12 and 24 h (Fig. 4b), whereas the effect on *miR-29b* was modest and highly variable (Supplementary Fig. 6a). The effect of IL-33 on *miR-29a* expression was NF-κB dependent (Supplementary Fig. 6b). Since IL-33-mediated collagen 3 matrix changes could be regulated by *miR-29a*, we analysed the functional effects of *miR-29a* manipulation on collagen matrix synthesis *in vitro*. Using luciferase assays, we first confirmed that *miR-29a* directly targets *COL 1A1* and *3A1* as previously demonstrated^28^ (Supplementary Fig. 6c). We also observed a previously unrecognized interaction of *miR-29a* with human *COL 1A2* subunit transcript (Fig. 4c). To test whether *miR-29a* indeed regulates the levels of candidate target mRNAs in disease relevant cells, we transfected tenocytes with *miR-29a* mimic and antagomir. *miR-29a* manipulation selectively regulated collagen 3 but not collagen 1 mRNA and protein expression in primary tenocytes (Fig. 4d,e). Moreover, *miR-29a* overexpression significantly decreased IL-33-induced collagen 3 mRNA and protein synthesis (Supplementary Fig. 6d and Fig. 4f). In additiona, *miR-29a* inhibition resulted in a significant increase in *COL 3A1* expression (Fig. 4g), indicating that *miR-29a* is not only actively regulating these transcripts in human tenocytes but its loss can be an important factor in the increase of type 3 collagen production observed in tendinopathy. In contrast, *COL 1A1 and A2* transcript levels were relatively unchanged (Fig. 4g).

Given that *miR-29a* was capable of repressing *COL 1A1* and *1A2* with equal or greater efficiency than collagen 3 in luciferase reporter assays, this was unlikely to be the result of *miR-29a* having greater affinity for its microRNA (miRNA) recognition element (MREs) in type 3 transcripts. One well-documented mechanism by which transcripts modulate their sensitivity to miRNA regulation is through the utilization of alternative polyadenylation signals (Fig. 4h)^29^. Use of distal polyadenylation signals results in reduction in 3′ untranslated region (UTR) length with the loss of MREs. To test this, we compared the levels of full-length (*miR-29a* MRE containing) *COL1A1*, *COL1A2* and *COL3A1* transcripts expressed in human tenocytes with their total levels by quantitative PCR (qPCR; Fig. 4i). We found that 95% of *COL1A1* and *1A2* transcripts did not contain *miR-29a* MRE, whereas the majority (∼75%) of *COL3A1* transcripts did. 3′ rapid amplification of complementary DNA (cDNA ends, RACE; Supplementary Fig. 6e) confirming that both *COL1A1* and *1A2* make use of previously unrecognized polyadenylation signals (Fig. 4h). Analysis of *COL3A1* 3′RACE products identified transcripts containing a single *miR-29a* MRE due to use of a proximal polyadenylation signal. *COL3A1* luciferase constructs, which contained only this MRE, were still efficiently repressed by *miR-29a* indicating that all *COL3A1* transcripts are sensitive to regulation by *miR-29a* (Supplementary Fig. 6f). Inspection of 3′RACE sequences revealed the presence of canonical polyadenylation signals upstream of polyA tails. The resulting truncated COL1A1 and 1A2 3′UTRs lack *miR-29a* MREs. These data suggest that in tenocytes *miR-29a* specifically regulates *COL3A1*, while both *COL1A1* and *COL1A2* are rendered insensitive to *miR-29a* inhibition due to the utilisation of alternative polyadenylation signals. Loss of *miR-29a* on IL-33 signalling results in the derepression of collagen 3 likely contributing to the increase of this collagen observed in injured tendons.

### Soluble ST2 is a direct target of *miR-29a*

**Figure 5:**
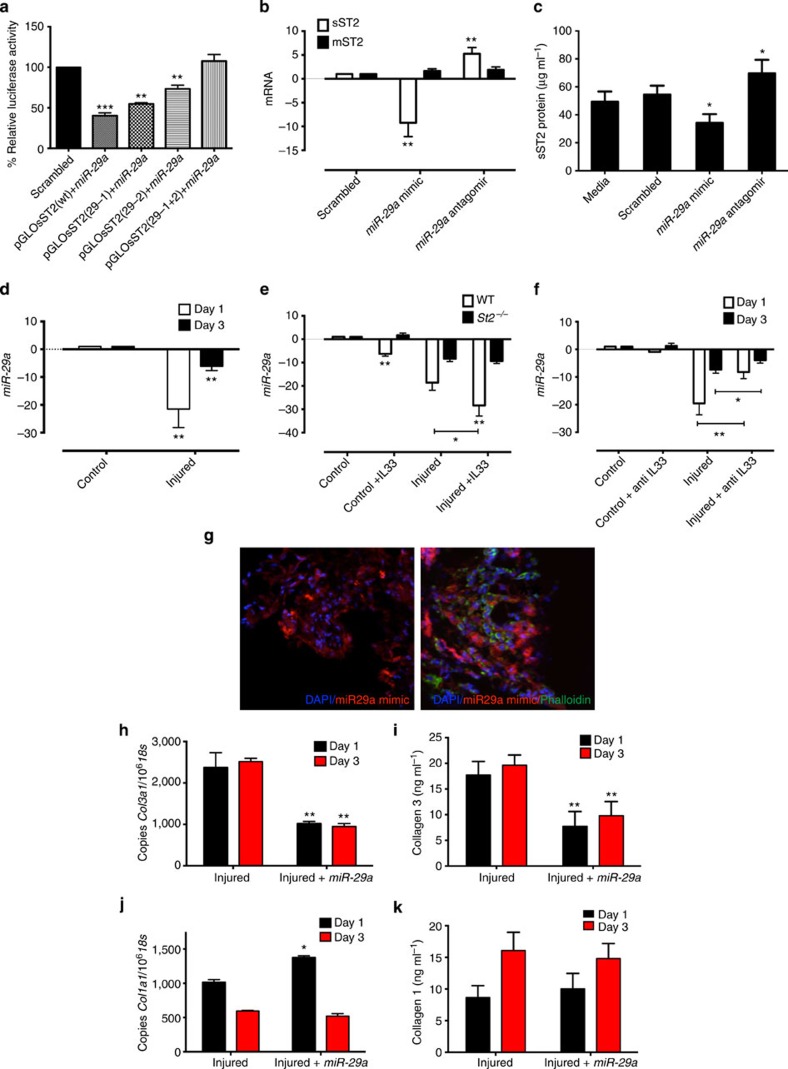
Crosstalk between IL-33/ST2 and *miR-29a*. (**a**) Luciferase activity assay of HEK 293 cells co-transferred with pre-*miR-29a* containing 3′UTR of WT or mutated soluble *ST2* and MREs of 3′UTR of soluble *ST2*. (*n*=3). **P<0.01, ***P<0.001 versus scrambled control. (Student's *t*-test). (**b**) *sST2* and *mST2* mRNA levels following addition of scrambled mimic, *miR-29a* mimic or *miR-29a* antagomir in human tenocyte cultures. (*n*=5), **P<0.01 versus scrambled control (Student's *t*-test). (**c**) sST2 protein production by tenocytes following incubation with *miR-29a* mimic/antagomir. (*n*=5), *P<0.05 versus scrambled control. (Student's *t*-test). (**d**) qPCR showing fold change in *miR-29a* in tendon of WT mice post injury. (**e**) qPCR showing fold change in *miR-29a* in WT and *St2*^*−/−*^ mice following treatment with rhIL-33 or PBS on Day 1 post injury. (**f**) *miR-29a* expression in the tendon of WT mice post injury with or without anti-IL-33 treatment. Data in **d**–**f** are mean fold change±s.d. of duplicate samples and are representative of four mice per group (*n*=16), *P<0.05, **P<0.01 versus control (ANOVA). Data for mRNA show expression relative to *u6* housekeeping gene. (**g**) Immunofluorescence stain of mimic (red) and counterstained with phalloidin (green, for cytoskeletal structure) showing localization of mimic around tenocytes at 24 h post injection of miR-29a mimic in WT mice. Scale bar, 80 nm. (**h**) *Col3* mRNA, (**i**) Collagen 3 protein, (**j**) *Col1* mRNA and (**k**) Collagen 1 protein levels post treatment with *miR-29a* mimic after tendon injury in WT mice. Data for mRNA are total copy number of gene versus 18S housekeeping gene in duplicate samples. Data in **h**–**k** are mean±s.d. of duplicate samples, representative of six mice per group, *P<0.05, **P<0.01 versus control (ANOVA).

Computational analysis revealed that *miR-22*, *183*, *25* and *29a* were predicted to regulate *sST2*. However, *miR-22*, *183* and *25* showed much less favourable probability in targeting *sST2* (total context score value^27^) than *miR-29* family. Importantly, only *miR-29* family members showed a good probability in targeting both collagens and *sST2*, thus suggesting a feasible regulatory role in IL-33 effector functions. A luciferase reporter gene was generated that contains the 3′UTR of human *sST2* predicted to possess two potential *miR-29abc*-binding sites (Supplementary Fig. 6g). Co-transfection of *sST2*-luciferase reporter plasmid with *miR-29* mimics in HEK 293 cells resulted in significant reduction in luciferase activity relative to scrambled control (Supplementary Fig. 6h). Furthermore, luciferase activity was fully restored when the seed regions of both *miR-29* MREs in *sST2* were mutated, demonstrating that *sST2* is a direct target of *miR-29a* (Fig. 5a). To investigate whether *miR-29a* does indeed regulate the levels of the candidate target mRNA in tenocytes, we again transfected *miR-29a* mimic or antagomir into human tenocytes. Soluble *ST2* message was significantly decreased by transfection with *miR-29a* mimic and increased by antagomir (Fig. 5b), with a corresponding significant change in soluble ST2 protein (Fig. 5c). These results confirm soluble *ST2* as a target of *miR-29a*. We also noted that *miR-29a* mimic transfection produced no effect on the production of rhIL-33-induced cytokines (IL-6, IL-8 and monocyte chemoattractant protein-1; Supplementary Fig. 6i), suggesting that *miR-29a* is not directly involved in the regulation of IL-33 canonical IL-1R signalling in tenocytes.

### IL-33/sST2 regulates *miR-29a* expression in tendon healing

We next investigated *miR-29a* expression in our *in vivo* tendon-healing model. Tendon injury in WT mice resulted in a 22-fold decrease in *miR-29a* on day 1, which reverted to a sixfold decrease (versus baseline) by day 3 (Fig. 5d and Supplementary Fig. 7a) with no significant difference by day 7. This effect was significantly decreased in *St2*^*−/−*^ mice (Supplementary Fig. 7a). In addition, the administration of exogenous rhIL-33 reduced *miR-29a* expression in uninjured tendons at all time points compared with PBS-injected controls (Supplementary Fig. 7b). This effect was most profound in injured WT mice, with the addition of rhIL-33 mediating a further 10-fold reduction in *miR-29a* (Fig. 5e). Addition of rhIL-33 in *St2*^*−/−*^ mice had no significant effect on *miR-29a* expression in injured or uninjured tendons (Fig. 5e), again suggesting that *miR-29a* downregulation is, at least in part, directly mediated by IL-33/ST2-dependent signalling. The addition of neutralizing antibody to IL-33 significantly reduced the effect of injury on *miR-29a* gene expression at days 1 and 3 post injury (Fig. 5f).

### Overexpression of *miR-29a* regulates collagen changes *in vivo*

Finally, based on our observations that the reduction of *miR-29a* function was linked to collagen matrix changes in human and animal models of tendon disease, we investigated the role of overexpression of *miR-29a in vivo* in an attempt to reverse these changes. Delivery of *miR-29a* mimic to the *in vivo* tendon-healing model, with tissue uptake confirmed by immunofluorescence and qPCR (Fig. 5g and Supplementary Fig. 7c), resulted in a significant reduction (*P*<0.01) of collagen 3 synthesis at days 1 and 3 post injury (Fig. 5h,i). While a transient increase in *Col1* message was noted at day 1 post injury (Fig. 5j), *miR-29a* overexpression caused no reduction in collagen 1 protein levels *in vivo* at days 1 and 3 post injury compared with control samples (Fig. 5k).

## Discussion

miRNAs have emerged as powerful regulators of diverse cellular processes with important roles in disease and tissue remodelling. Studies reported here using tendinopathy as a model system reveal the previously unrecognized ability of a single miRNA (*miR-29a*) to cross-regulate inflammatory cytokine effector function and extracellular matrix in the complex early biological processes leading to tissue repair. We also provide evidence for a hitherto unreported role of IL-33 in the initial steps that lead to the important clinical entity of tendinopathy.

IL-33 has recently become increasingly associated with musculoskeletal pathologies^16^. Our data show IL-33 to be present in human tendon biopsies at the early stage of disease, while end-stage biopsies have significantly less IL-33 expression at the message and protein level supporting the concept of IL-33 as an early tissue mediator in tendon injury and subsequent tissue remodelling. On cell injury, the so-called alarmins such as heat shock proteins^30^, HMGB1 (ref. 31), uric acid^32^ and IL-1 family members^33^,^34^, including IL-33 (refs 35, 36), are released by both resident immune cells and necrotic cells having undergone damage. These alarmins are subsequently recognized by various immune cells that initiate inflammatory and repair responses. Our data implicate IL-33 as an alarmin in early tendinopathy and, importantly, our biomechanical data suggest that such an expression has a pathogenically relevant role. The addition of rhIL-33 significantly reduced the tendon strength (load to failure) of WT mice by ∼30% at early time points, likely as a consequence of the concomitant collagen 3 matrix changes, which result in mechanically inferior tendon^37^. Thus, one plausible mechanism for the events, mediating early biomechanically inferior tendon repair, is that on repeated micro injury IL-33 is upregulated with its subsequent release through mechanical stress/necrosis. IL-33 then drives the matrix degeneration and proinflammatory cytokine production, propelling the tendon towards a pathological state such as that seen in early tendinopathy biopsies. Interestingly, the addition of IL-33-neutralizing antibodies to injured mice reversed the collagen 3 synthesis, but this was only able to temporarily improve tendon strength on day 1 post injury. While this may negate blocking IL-33 in longer-term sports injuries, the repetitive microtrauma associated with pathological tendon changes may conversely allow neutralizing IL-33 to act as a check rein to further unwanted matrix dysregulation. Although these results do not exclude the contribution of other immune cells to IL-33-mediated tendon pathology, our *in vitro* studies performed on tenocytes show a robust effect of IL-33 on the differential collagen 1:3 ratio, which is replicated in the *in vivo* setting. Thus, while immune cells will undoubtedly be relevant to tendon damage *in vivo,* our results support the notion that tenocytes are of substantial significance in the IL-33-mediated changes in tendon injury.

**Figure 6:**
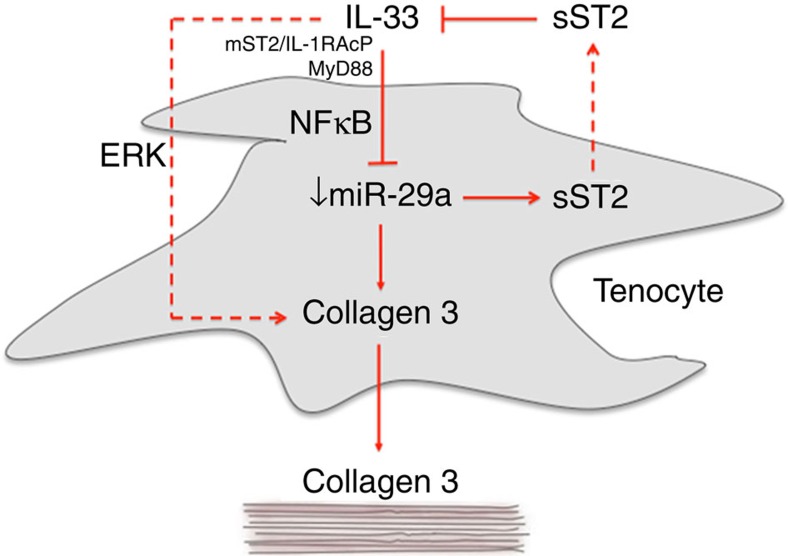
Schematic representation of the role of IL-33 and miR-29a in tendon pathology. Tendon injuries or repetitive micro tears causing stress of tendon cells result in the release of IL-33 from tenocytes or immune cells and the downstream phosphorylation of NFκB which in turn represses *miR-29a* causing an increase in collagen type 3 and sST2 production. IL-33 may also increase collagen synthesis in an ERK-dependent manner. The increased presence of collagen 3 reduces the tendon tensile strength lending to early tendon failure. However, elevated sST2 may act, in a feedback loop fashion, as a protective mechanism by removing excess IL-33 from the system.

Emerging studies highlight miRNAs as key regulators of leukocyte function and the cytokine network while orchestrating proliferation and differentiation of stromal lineages that determine extracellular matrix composition^38^. The finding of a key role for *miR-29a* in the regulation of IL-33 ‘alarmin'-mediated effects provides mechanistic insight into miRNA cross-regulatory networks involving inflammation and matrix regulation in tissue repair. Our data demonstrate a functional role for *miR-29a* as a post-transcriptional regulator of collagen in murine and human tendon injury. The regulation of collagens and other extracellular matrices by the *miR-29* family has been highlighted in several prior studies^28^,^29^,^39^,^40^,^41^. Our results now suggest that *miR-29a* acts as a critical repressor to regulate collagen expression in tendon healing. Moreover, the reduced expression of *miR-29a* in human biopsies suggests that its functional diminution leads to development of tendinopathy. Despite tendon pathology being characterized by increased collagen 3 deposition resulting in biomechanical inferiority and degeneration, the molecular premise for this collagen ‘switch' has hitherto been unknown. We describe here that IL-33-induced deficiency in *miR-29a* results in an overproduction of collagen 3 while simultaneously setting in motion, via sST2 inhibition of IL-33, the ultimate resolution of this early repair process (Fig. 6). Contrary to expectations in human tenocytes, *miR-29a* was only capable of influencing the expression of *COL3A1* and not type 1 collagens. Subsequent characterization of the 3′UTR of type 1 and 3 collagens revealed a previously unreported pattern of alternative polyadenylation in both type 1 subunits, resulting in transcripts lacking *miR-29a*-binding sites rendering them insensitive to repression by this miRNA. This was not the case for type 3 collagen transcripts, which retain *miR-29a*-binding sites. In human tenocytes, collagen 3 is actively repressed by *miR-29a*, as demonstrated by the ability of *miR-29a* inhibitors to significant increase collagen 3 levels. Furthermore, supplementing tenocytes with *miR-29a* in the presence of IL-33 was sufficient to inhibit the increased production of collagen 3. These results indicate that *miR-29a*-mediated regulation of collagen 3 transcript stability is the predominant factor in the observed increased collagen 3 production. Importantly, in our model system, *miR-29a* additionally targeted the IL-33 decoy receptor sST2. Thus, IL-33-driven loss of *miR-29a* expression results in the simultaneous repression of collagen 3 and sST2, with a subsequent autoregulatory inhibition of IL-33 promoting the resolution of the immediate alarmin response. Alternative miRNAs have been described at various stages of the tendinopathy spectrum^42^,^43^. While it is likely that *miR29a* targets other important genes^44^,^45^ in the complex tendon inflammatory/matrix mileu, our data suggest that the reintroduction of *miR-29a* to the injury-induced *miR-29a* deficiency in tendon results in reversal of the key collagen switch that remains a core pathological feature of tendinopathy linked to ultimate clinical tendon rupture.

On the basis of this work, we propose IL-33 as an influential alarmin in the unmet clinical area of early tendon injury and tendinopathy, which may be important in the balance between reparation and degeneration. A previously unrecognized role for *miR-29a* as a post-transcriptional regulator of matrix/inflammatory genes in tendon healing and tendinopathy has been uncovered. One of the great promises of exploiting miRNAs for therapeutic purposes has been the potential of a single miRNA to regulate functionally convergent target genes. Our discovery of a single miRNA-dependent regulatory pathway in early tissue healing highlights *miR-29a* replacement therapy as a promising therapeutic option for tendinopathy with implications for other human pathologies in which matrix dysregulation is implicated.

## Methods

### Human model of tendinopathy

All procedures and protocols were approved by the Ethics Committee under ACEC No. 99/101 with informed consent obtained as per standard operative procedures. Seventeen supraspinatus tendon (torn tendon representing established pathology) samples (sample size based on our previous findings of significance with 10–12 patient samples^46^,^47^) were collected from patients with rotator cuff tears undergoing shoulder surgery (Supplementary Table 1). The mean age of the rotator cuff ruptured patients was 54 years (range, 35–70 years)—the mean tear size was 2.5 cm. Samples of the subscapularis tendon (representing ‘early pathology') were also collected from the same patients. Patients were only included if there was no clinically detectable evidence of subscapularis tendinopathy on a preoperative MRI scan or macroscopic damage to the subscapularis tendon at the time of arthroscopy—by these criteria, they represented a truly preclinical cohort. An independent control group was obtained comprising 10 samples of subscapularis tendon collected from patients undergoing arthroscopic surgery for shoulder stabilization without rotator cuff tears. The absence of rotator cuff tears was confirmed by arthroscopic examination. The mean age of the control group was 35 years (range, 20–41 years). All torn supraspinatus samples showed Grade 4 changes consistent with marked degeneration, mucoid change and frank chondroid metaplasia. Matched subscapularis tendon showed Grade 2–3 changes indicative of moderate-advanced tendinopathy. All control samples were classified as Grade 1 consistent with normal fibrotendinous tissue with large distinct collagen fibrils. There were no significant correlations between Bonar score and the mean duration of symptoms or age of the patient cohort.

### Tissue collection and preparation

Arthroscopic repair of the rotator cuff was carried out using the standard three-portal technique and the cross-sectional size of the rotator cuff tear was estimated and recorded as described previously^48^. The subscapularis tendon was harvested arthroscopically from the superior border of the tendon 1 cm lateral to the glenoid labrum. The supraspinatus tendon was harvested from within 1.5 cm of the edge of the tear before surgical repair. For immunohistochemical staining, the tissue samples were immediately fixed in 10% (v/v) formalin for 4 to 6 h and then embedded in paraffin. Sections were cut to 5 μm thickness using a Leica-LM microtome (Leica Microsystems, Germany) and placed onto Superfrost Ultra Plus glass slides (Gerhard Menzel, Germany). Paraffin was then removed from the tissue sections with xylene, rehydrated in graded alcohol and used for histological and immunohistochemical staining according to previously established methodologies^49^.

Human tendon-derived cells were explanted from hamstring tendon tissue of five patients (aged 18–30 years) undergoing hamstring tendon anterior cruciate ligament reconstruction. Normal tendinous structure and Bonar scoring was ensured by histology before inclusion. Cultures were maintained at 37 °C in a humidified atmosphere of 5% CO_2_ for 28 days. Cells were subcultured and trypsinized at subconfluency. Cells from the third and fourth passage were used and tenocyte lineage was confirmed by tenascin C, CD55 and scleraxis staining as per our previous protocols^46^.

We recognize the limitations of anatomical, environmental and biomechanical differences between rotator cuff and hamstring tendons. However, our rationale behind using these models is based on our previous reports utiliszng the same systems^23^,^46^ and ultimately, within the field, they remain the ‘gold standard ‘ for allowing comparative molecular investigation of the early events in tendon injury/disease^50^.

### Histology and immunohistochemistry

Human tendon sections were stained with haematoxylin and eosin and toluidine blue for determination of the degree of tendinopathy as assessed by a modified version of the Bonar score^51^ (Grade 4, marked tendinopathy; 3, advanced tendinopathy; 2, moderate degeneration; 1, mild degeneration; 0, normal tendon). This included the presence or absence of oedema and degeneration together with the degree of fibroblast cellularity and chondroid metaplasia. Thereafter, the sections were stained with antibodies directed against the following markers:—IL-33 (Alexis ALX-804–840, mouse monoclonal 5 μg ml^−1^), ST2 (R&D Systems, Europe, Clone 97203, mouse monoclonal 15 μg ml^−1^), IL-1RaCP (ProSci 2131, rabbit polyclonal 5 μg ml^−1^), CD68 (pan macrophages, clone KP-1, Vector 1 μg ml^−1^), CD3 (T cells, cone LN10, Vector Labs 1.25 μg ml^−1^), CD4 (T helper cells clone 1F6, Dako Labs 1 μg ml^−1^), CD206 (M2 macrophages, R&D Systems, AF2534 5 μg ml^−1^) and mast cell tryptase (Mast cells clone 10D11, Vector Labs 1 μg ml^−1^).

Endogenous peroxidase activity was quenched with 3% (v/v) H_2_O_2_, and nonspecific antibody binding blocked with 2.5% horse serum in TBST (Tris-buffered saline with Tween 20) buffer for 30 min. Antigen retrieval was performed in 0.01 M citrate buffer for 20 min in a microwave. Sections were incubated with primary antibody in 2.5% (w/v) horse serum/human serum/TBST at 4 °C overnight. After two washes, the slides were incubated with Vector ImmPRESS Reagent kit as per the manufacturer's instructions for 30 min. The slides were washed and incubated with Vector ImmPACT DAB chromagen solution for 2 min, followed by extensive washing. Finally, the sections were counterstained with haematoxylin. Positive (human tonsil tissue) specimens were included, in addition to the surgical specimens for each individual antibody staining technique. Omission of primary antibody and use of negative control isotype confirmed the specificity of staining.

We applied a scoring system based on previous methods^47^ to quantify the immunohistochemical staining. Ten random high power fields ( × 400) were evaluated by three independent assessors (N.L.M., J.H.R., A.L.C.). In each field, the number of positive and negatively stained cells were counted and the percentage of positive cells calculated giving the following semi-quantitative grading; Grade 0, no staining; Grade 1, <10% cells stained positive; 2, 10–20% cells stained positive; Grade 3, >20% cells positive.

Mouse tendon sections were processed using the above protocol with antibodies directed against the following markers:—IL-33 (R&D systems AF3626, mouse monoclonal), ST2 (R&D Systems Clone 97203, mouse monoclonal 15 μg ml^−1^) and F4/80 (Serotec clone CI:A3-1, mouse monoclonal 20 μg ml^−1^)).

### Matrix regulation

Total soluble collagen was measured from cell culture supernatants using the Sircol assay kit (Biocolor, Carrickfergus, Northern Ireland) according to the manufacturer's protocol. Sircol dye reagent (1 ml) was added to test sample (100 μl) and mixed for 30 min at room temperature. The collagen–dye complex was precipitated by centrifugation at 10,000*g* for 10 min and then washed twice with 500 μl of ethanol. The pellet was dissolved in 500 μl of alkali reagent. Absorbance was measured by microplate reader at 540 nm, with standard provided by the manufacturer. The concentrations of human and mouse collagen 1 and 3 were assessed using ELISA (USCNK Life Science Inc) with colour change measured at 450 nm by microplate reader, along with standards supplier by the manufacturer.

### Signalling experiments

Phosphorylation status of MAPKs (mitogen-activated protein kinases), extracellular signal regulated kinases (ERK1/2), c-Jun N-terminal kinases (JNKs) and p38 isoforms were evaluated using the Human Phospho-MAPK Array (R&D Systems) as per the manufacturer's instructions. The ERK inhibitor (FR180204) was purchased from CalbioChem (Merck KGaA, Germany) and used at IC_50_=10 μM, a concentration previously determined to offer optimal specific inhibition relative to off target effects, which was used previously in our laboratory^52^.

Phosphorylation of NF-κB p65 was assessed using the InstantOne ELISA in cell lysates from treated and untreated tenocytes. Absorbance was measured at 450 nm by microplate reader with positive and negative controls supplied by the manufacturer. The relative absorbance of stimulated versus unstimulated cells was used to assess the total or phosphorylated NF-κB p65 in each sample. The NF-κB inhibitor (SN50) was purchased from CalbioChem (Merck KGaA, Germany) and used at IC_50_=11 nM.

### RNA extraction and quantitative PCR

The cells isolated from experiments were placed in Trizol before mRNA extraction. QIAgen mini columns (Qiagen) were used for the RNA clean-up with an incorporated on column DNAse step as per the manufacturer's instructions. cDNA was prepared from RNA samples according to AffinityScript (Agilent Technologies) multiple temperature cDNA synthesis kit as per the manufacturer's instructions. Real-time PCR was performed using SYBR green or Taqman FastMix (Applied Biosystems,) according to whether a probe was used with the primers. The cDNA was diluted 1 in 5 using RNase-free water. Each sample was analysed in triplicate. Primers (Integrated DNA Technologies, Belgium) were as follows: human *GAPDH*, 5′-TCG ACA GTC AGC CGC ATC TTC TTT-3′ (f) and 5′-ACC AAA TCC GTT GAC TCC GAC CTT-3′ (r); mouse *Gapdh* 5′-TGG CAA AGT GGA GAT TGT TGC C-3′ (f) and 5′-AAG ATG GTG ATG GGC TTC CCG-3′ (r); human *18S*, 5′-GTA ACC CGT TGA ACC CCA TT-3′ (f) and 5′-CCA TCC AAT CGG TAG TAG CG -3′ (r); mouse *18s*, 5′-TTG ACG GAA GGG CAC CAC CAG-3′ (f) and 5′-CTC CTT AAT GTC ACG CAC GAT TTC-3′ (r); human *U6*, 5′-GTG CTC GCT TCG GCA GCA CAT ATA C-3′ (f) and 5′-AAA AAT ATG GAA CGC TTC ACG AAT TTG-3′ (r); human *IL33*, 5′-GGA AGA ACA CAG CAA GCA AAG CCT-3′ (f) and 5′-TAA GGC CAG AGC GGA GCT TCA TAA-3′ (r); murine *Il33*, 5′-GGA AGA ACA CAG CAA GCA AAG CCT-3′ (f) and 5′-TAA GGC CAG AGC GGA GCT TCA TAA-3′ (r); total human *ST2*, 5′-ACA ACT GGA CAG CAC CTC TTG AGT-3′ (f) and 5′-ACC TGC GTC CTC AGT CAT CAC ATT-3′ (r); murine *sSt2*, 5′-CCA ATG TCC CTT GTA GTC GG (f) and CTT GTT CTC CCC GCA GTC (r), TCC CCA TCT CCT CAC CTC CCT TAA T-3′ (probe); mouse *mSt2*, 5′-TCT GCT ATT CTG GAT ACT GCT TTC-3′ (f) and 5′-TCT GTG GAG TAC TTT GTT CAC C-3′ (r), 5′-AGA GAC CTG TTA CCT GGG CAA GAT G-3′ (probe); human *mST2*, 5′-ACA AAG TGC TCT ACA CGA CTG-3′ (f) and 5′-TGT TCT GGA TTG AGG CCA C-3′ (r); 5′-CCC CAT CTG TAC TGG ATT TGT AGT TCC G-3′ (probe); human *sST2*, 5′-GAG ACC TGC CAC GAT TAC AC-3′ (f) and 5′-TGT TAA ACC CTG AGT TCC CAC-3′ (r), 5′-CCC CAC ACC CCT ATC CTT TCT CCT-3′ (probe); human *COL3A*, 5′-TTG GCA GCA ACG ACA CAG AAA CTG-3′ (f) and 5′-TTG AGT GCA GGG TCA GCA CTA CTT-3′ (r); mouse *Col3a*, 5′-GCT TTG TGC AAA GTG GAA CCT GG-3′ (f) and 5′-CAA GGT GGC TGC ATC CCA ATT CAT-3′ (r); human *COL1A1*, 5′-CCA TGC TGC CCT TTC TGC TCC TTT-3′ (f) and 5′-CAC TTG GGT GTT TGA GCA TTG CCT-3′ (r); mouse *Col 1a1*, 5′-TTC TCC TGG CAA AGA CGG ACT CAA-3′ (f) and 5′-GGA AGC TGA AGT CAT AAC CGC CA-3′ (r). All mRNA and miRNA data sets (except Fig. 4a) represent fold change in gene expression compared with designated control utilizing housekeeping genes *GAPDH*, *18S* or *U6* as detailed in Figure legend. Figure 4a shows threshold cycle (*C*t) data for *miR-29a/b/c* expression in human tenocytes and human tendon tissue samples. Lower *C*t value represents higher message abundance.

### RNA isolation and quantitative PCR of miRNA

Total RNA was isolated by miRNeasy kit (Qiagen). miScript Reverse Transcription Kit (Qiagen) was used for cDNA preparation. TaqMan mRNA assays (Applied Biosystems) or miScript primer assay (Qiagen) were used for quantitative determination of the expression of human *miR-29a* (MS00001701) *miR-29b* (MS00006566) and *miR-29c* (MS00009303) and mouse *miR-29a* (MS00003262), *miR-29b* (MS00005936) and *miR-29c* (MS00001379). Expressions of *U6B* small nuclear RNA or β-actin were used as endogenous controls.

### Quantification of alternative polyadenylated collagen transcripts

The absolute levels of long and short 3′UTR forms of type I and III transcripts were determined by qPCR relative to standards. cDNA was generated using AffinityScript (Agilent) with both random hexamer and oligo-dT primers. SYBR green qPCR was performed using the following primers: Samples were normalized to *GAPDH* endogenous control.

COL1A2_S FW 5′-GCCTGCCCTTCCTTGATATT-3′

COL1A2_S REV 5′-TGAAACAGACTGGGCCAATG-3′

COL1A2_L FW 5′-TCAGATACTTGAAGAATGTTGATGG-3′

COL1A2_L REV 5′-CACCACACGATACAACTCAATAC-3′

COL1A1_S FW 5′-CTTCACCTACAGCGTCACT-3′

COL1A1_S REV 5′-TTGTATTCAATCACTGTCTTGCC-3′

COL1A1_L FW 5′-CCACGACAAAGCAGAAACATC-3′

COL1A1_L REV 5′-GCAACACAGTTACACAAGGAAC-3′

COL3A1_S FW 5′-CTATGACATTGGTGGTCCTGAT-3′

COL3A1_S REV 5′-TGGGATTTCAGATAGAGTTTGGT-3′

COL3A1_L FW 5′-CCACCAAATACAATTCAAATGC-3′

COL3A1_L REV 5′-GATGGGCTAGGATTCAAAGA-3′

### 3′RACE

3′RACE was performed on RNA that had been reverse transcribed using AffinityScript (Agilent) by sequence tagged oligo-dT primers (QT, Q0 Q1). 3′ sequences were then amplified using nest-PCR approach with gene-specific primers (GSP 1/2). Amplified products were cloned into pCR2.1TOPO (Invitrogen) and sequenced.

QT 5′-CCAGTGAGCAGAGTGACGAGGACTCGAGCTCAAGCTTTTTTTTTTTTTTTTT-3′

Q0 5′-CCAGTGAGCAGAGTGACG-3′

Q1 5′-GAGGACTCGAGCTCAAGC-3′

COL1A1 GSP1 5′-CTTCCTGTAAACTCCCTCCATC-3′

COL1A1 GSP2 5′-AACAGACAAGCAACCCAAAC-3′

COL1A2 GSP1 5′-CAGACAAGCAACCCAAACTG-3′

COL1A2 GSP2 5′-AATGGGAGACAATTTCACATGG-3′

COL3A1 GSP1 5′-GCCTGCCCTTCCTTGATATT-3′

COL3A1 GSP2 5′-CCTTCCATTTCTTCTGCACATC-3′

### miRNA transfection

Cells (primary tenocytes) were transfected with synthetic mature miRNA for *miR-29a* and *b* or with negative control (*C. elegans miR-67* mimic labelled with Dy547, Thermo Scientific Inc.) at a final concentration of 20 nM with the use of Dharmacon DharmaFECT 3 siRNA transfection reagents (Thermo Scientific Inc.). At 48 h after transfection, the cellular lysates were collected to analyse the expression of genes of interest.

Transfection efficiency (80%) was assessed by flow cytometry using the labelled Dy547 mimic (Supplementary Fig. 7g) and confirmed by qPCR of control-scrambled mimic and the respective miR29 family mimic.

### Luciferase reporter assay for targeting collagen 1 and 3 and soluble ST2

The human 2 miRNA target site was generated by annealing the oligos: for *COL* 1 and 3 and soluble *ST2* 3′UTRs that were cloned in both sense and antisense orientations downstream of the luciferase gene in pMIR-REPORT luciferase vector (Ambion). These constructs were sequenced to confirm inserts and named pMIR-*COL I/COL III/ sST2*-*miR29a/b/c* and pMIR(A/S)-*COL I/COL III/ sST2*-*miR29a/b/c*, and used for transfection of HEK293 cells (HEK293 cells tested negative for mycoplasma before use). HEK293 cells were cultured in 96-well plates and transfected with 0.1 μg of either pMIR-*COL I/COL III/ sST2*-miR29a/b/c, pMIR(A/S)-*COL I/COL III/ sST2*-miR29a/b/c or pMIR-REPORT, together with 0.01 μg of pRL-TK vector (Promega) containing *Renilla* luciferase and 40 nM of *miR-29a* or scrambled miRNA (Thermo Scientific Dharmacon). Transfections were performed using Effectene (Qiagen) according to the manufacturer's instructions. Luciferase activity was measured 24 h after transfection using the Dual-Luciferase Reporter Assay (Promega). The 3′UTR of human *sST2* was amplified from genomic DNA using the following primers (f) 5′-AGTTTAAACTGGCTTGAGAAGGCACACCGT-3′ and (r) 5′-AGTCGACGGGCCAAGAAAGGCTCCCTGG-3′, which created PmeI and SalI sites, respectively. These sites where used to clone the PCR-amplified product into the same sites of pmiRGLO (Promega). The seed regions of the two Targetscan predicted *miR-29a* MRE sites: 29-1 and 29-2 were mutated using the QuickChange site-directed mutagenesis kit (Agilent). Each vector along with *miR-29a* or scrambled control mimic were transfected into HEK293 cells using Attactene (Qiagen) according to the manufacturer's instructions. Luciferase activity was measured 24 h later using Dual-Glo luciferase assay (Promega) with luciferase activity being normalized to Renilla. Normalized luciferase activity was expressed as a percentage of scrambled control for the same constructs.

### Cytokine production

Cytokine concentration in the supernatant of cultured human tenocytes was determined by 25-Plex human cytokine assay (with 25 separate human cytokines) using Luminex (Luminex Corp).

### Animals and patellar tendon injury model

BALB/c mice were purchased from Charles River. *St2*^*−/−*^ mice (of BALB/c background) were originally provided by Dr Andrew McKenzie (LMB, Cambridge). Mice were maintained at the Central Research Facility, University of Glasgow. All animal experimentation and husbandry were according to the UK Government Home Office Project License. All protocols were approved by the Glasgow University local Ethical Review Panel. Mice were used at 10–12 weeks old and were age matched for each independent experiment. *In vivo* experimental protocols are depicted in Supplementary Fig. 7e,f,h).

In preparation for the surgical procedure, mice were anaesthetized with a mixture of isofluorane (3%) and oxygen (1%) and both hind limbs were shaved. During surgical procedures, anaesthesia was delivered via a nose cone with the level of isofluorane reduced to 1% with oxygen. Following a skin incision, two cuts parallel to the tendon were made in the retinaculum on each side, a set of flat faced scissors were then placed underneath the patellar tendon. With the scissor blades serving as a support, a 0.75-mm diameter biopsy punch (World Precision Instruments) was used to create a full thickness partial transection in the right patellar tendon. The left patellar tendon underwent a sham procedure, which consisted of only placing the plastic backing underneath the tendon without creating and injury. The skin wounds were closed with skin staples and the mice were killed at day 1, 3, 7 or 21 post surgery. Mice were killed by CO_2_ inhalation and immediately weighted. Each group contained 16 mice (*n*=8, *St2*^*−/−*^ and 8 WT) per time point. These experiments were repeated on four separate occasions.

To test if IL-33 induced tendon matrix dysregulation, a cytokine injection model was used. IL-33 was tested in a previously reported model initially described for the application of IL-23 or IL-22 (refs 53, 54). *St2*^*−/−*^ mice (*n*=4 per group/treatment/experiment) were injected intraperitoneally daily with IL-33 (0.4 μg per mouse diluted in 100 μl PBS) on days *−*3, *−*2, *−*1 and the day of injury (day 0). Mice were culled 24 h after the final injection as per protocol. Control mice similarly received an equal volume of PBS. We also tested neutralizing antibodies to IL-33 (0.5 μg ml^−1^, R&D systems) by injecting (i.p) the antibody or normal IgG control immediately post injury in WT and *St2*^*−/−*^ mice (four per group/treatment/experiment).

*miR-29a* mimic (50 ng ml^−1^ in 0.1 ml) was delivered via direct injection to injured (immediately post injury) and uninjured mice tendons (*n*=6 per group/treatment/experiment). Control mice similarly received an equal volume of PBS. During these procedures, the GCP principles were applied. All samples were labelled in a numerical fashion and were then blinded during all further experimental methodology. Numerical codes were only revealed post experiment and analysis.

### Biomechanical analysis

For biomechanical analysis, the patellar tendons of mice from each group were injured and eight mice killed at one of the four time points for mechanical testing as described previously by Lin *et al.*^10^ (Supplementary Fig. 7f). On the basis of previous experiments, a sample size of eight per group offered 95% confidence interval that a 50% difference between injured and control littermate animals would be detected.

In brief, the patellar tendons were dissected and cleaned, leaving only the patella, patellar tendon and tibia as one unit. Tendon width and thickness were then quantified and cross-sectional area was calculated as the product of the two. The tibia was then embedded in Isopon p38 (High Build Cellulose Filler) in a custom-designed fixture and secured in place in a metal clamp. The patella was held in place by vice grips used with the BOSE ElectroForce 3200 test instrument. Each tendon specimen underwent the following protocol immersed in a 37 °C saline bath—reloaded to 0.02 N, preconditioned for 10 cycles from 0.02 to 0.04 at a rate of 0.1% s^−1^ (0.003 mm s^−1^), and held for 10 s. Immediately, a stress relaxation experiment was performed by elongating the tendon to a strain of 5% (0.15 mm) at a rate of 25% (0.75 mm/s), followed by a relaxation for 600 s. Finally, a ramp to failure was applied at a rate of 0.1% s^−1^ (0.003 mm s^−1^). From these tests, maximum stress was determined and modulus was calculated using linear regression from the near linear region of the stress strain curve.

### Statistical analysis

All results are shown as mean±s.d. and all statistical analysis was performed using Student's *t*-test, ANOVA (analysis of variance) test or Mann–Whitney test *U*-test, as indicated in figure legends, using the Graph Pad Prism 5 software. A *P* value of <0.05 was considered statistically significant.

## Author contributions

N.L.M., D.S.G., F.Y.L., M.K.-S. and I.B.M. conceived and designed the experiments. N.L.M., D.S.G., M.A., M.K.-S., J.H.R., S.C.K. and A.L.C. performed the experiments. G.A.C.M., F.Y.L. and I.B.M. provided expert advice. All authors analysed the data. N.L.M., D.S.G., F.Y.L., M.K.-S., I.B.M. wrote the paper.

## Additional information

**How to cite this article:** Millar, N. L. *et al.* *MicroRNA29a* regulates IL-33-mediated tissue remodelling in tendon disease. *Nat. Commun.* 6:6774 doi: 10.1038/ncomms7774 (2015).

## Supplementary Material

Supplementary Information Supplementary Figures 1-7, Supplementary Table 1

## References

[b1] Eming S.A., Krieg T. & Davidson J.M. Inflammation in wound repair: molecular and cellular mechanisms. J. Invest. Dermatol. 127, 514–525 (2007) . 17299434 10.1038/sj.jid.5700701

[b2] McCormick A., Charlton J. & Fleming D. Assessing health needs in primary care. Morbidity study from general practice provides another source of information. BMJ 310, 1534 (1995) . 7787617 10.1136/bmj.310.6993.1534d PMC2549904

[b3] Khan K. M., Cook J. L., Bomar F., Harcourt P. & Astrom M. Histopathology of common tendinopathies. Update and clinical management Sports Med 27, 393–408 (1999) . 10.2165/00007256-199927060-00004 10418074

[b4] Sharma P. & Maffulli N. Tendon injury and tendinopathy: healing and repair. J. Bone Joint Surg. Am. 87, 187–202 (2005) . 15634833 10.2106/JBJS.D.01850

[b5] Millar N. L., Wei A. Q., Molloy T. J., Bonar F. & Murrell G. A. Cytokines and apoptosis in supraspinatus tendinopathy. J. Bone Joint Surg. Br. 91, 417–424 (2009) . 19258623 10.1302/0301-620X.91B3.21652

[b6] Pufe T., Petersen W., Tillmann B. & Mentlein R. The angiogenic peptide vascular endothelial growth factor is expressed in foetal and ruptured tendons. Virchows Arch. 439, 579–585 (2001) . 11710646 10.1007/s004280100422

[b7] Tsuzaki M. *et al.* IL-1 beta induces COX2, MMP-1, -3 and -13, ADAMTS-4, IL-1 beta and IL-6 in human tendon cells. J. Orthop. Res. 21, 256–264 (2003) . 12568957 10.1016/S0736-0266(02)00141-9

[b8] Tohyama H., Yasuda K., Uchida H. & Nishihira J. The responses of extrinsic fibroblasts infiltrating the devitalised patellar tendon to IL-1beta are different from those of normal tendon fibroblasts. J. Bone Joint Surg. Br. 89, 1261–1267 (2007) . 17905970 10.1302/0301-620X.89B9.18053

[b9] John T. *et al.* Effect of pro-inflammatory and immunoregulatory cytokines on human tenocytes. J. Orthop. Res. 28, 1071–1077 (2010) . 20127972 10.1002/jor.21079

[b10] Lin T. W., Cardenas L., Glaser D. L. & Soslowsky L. J. Tendon healing in interleukin-4 and interleukin-6 knockout mice. J. Biomech. 39, 61–69 (2006) . 16271588 10.1016/j.jbiomech.2004.11.009

[b11] Gulotta L. V., Kovacevic D., Cordasco F. & Rodeo S. A. Evaluation of tumor necrosis factor alpha blockade on early tendon-to-bone healing in a rat rotator cuff repair model. Arthroscopy 27, 1351–1357 (2011) . 21705172 10.1016/j.arthro.2011.03.076

[b12] Schmitz J. *et al.* IL-33, an interleukin-1-like cytokine that signals via the IL-1 receptor-related protein ST2 and induces T helper type 2-associated cytokines. Immunity 23, 479–490 (2005) . 16286016 10.1016/j.immuni.2005.09.015

[b13] Lefrancais E. *et al.* IL-33 is processed into mature bioactive forms by neutrophil elastase and cathepsin G. Proc. Natl Acad. Sci. USA 109, 1673–1678 (2012) . 22307629 10.1073/pnas.1115884109 PMC3277172

[b14] Kakkar R., Hei H., Dobner S. & Lee R. T. Interleukin 33 as a mechanically responsive cytokine secreted by living cells. J. Biol. Chem. 287, 6941–6948 (2012) . 22215666 10.1074/jbc.M111.298703 PMC3307313

[b15] Lamkanfi M. & Dixit V. M. IL-33 raises alarm. Immunity 31, 5–7 (2009) . 19604486 10.1016/j.immuni.2009.06.011

[b16] Liew F. Y., Pitman N. I. & McInnes I. B. Disease-associated functions of IL-33: the new kid in the IL-1 family. Nat. Rev. Immunol. 10, 103–110 (2010) . 20081870 10.1038/nri2692

[b17] Asirvatham A. J., Magner W. J. & Tomasi T. B. miRNA regulation of cytokine genes. Cytokine 45, 58–69 (2009) . 19121586 10.1016/j.cyto.2008.11.010 PMC3129852

[b18] Pritchard C. C., Cheng H. H. & Tewari M. MicroRNA profiling: approaches and considerations. Nat. Rev. Genet. 13, 358–369 (2012) . 22510765 10.1038/nrg3198 PMC4517822

[b19] Xu D. *et al.* IL-33 exacerbates antigen-induced arthritis by activating mast cells. Proc. Natl Acad. Sci. USA 105, 10913–10918 (2008) . 18667700 10.1073/pnas.0801898105 PMC2491487

[b20] Zaiss M. M. *et al.* IL-33 shifts the balance from osteoclast to alternatively activated macrophage differentiation and protects from TNF-alpha-mediated bone loss. J. Immunol. 186, 6097–6105 (2011) . 21515798 10.4049/jimmunol.1003487

[b21] Rankin A. L. *et al.* IL-33 induces IL-13-dependent cutaneous fibrosis. J. Immunol. 184, 1526–1535 (2010) . 20042577 10.4049/jimmunol.0903306

[b22] Matthews T. J., Hand G. C., Rees J. L., Athanasou N. A. & Carr A. J. Pathology of the torn rotator cuff tendon. Reduction in potential for repair as tear size increases. J. Bone Joint Surg. Br. 88, 489–495 (2006) . 16567784 10.1302/0301-620X.88B4.16845

[b23] Millar N. L. *et al.* Inflammation is present in early human tendinopathy. Am. J. Sports Med. 38, 2085–2091 (2010) . 20595553 10.1177/0363546510372613

[b24] Ogawa T. *et al.* Suppression of type I collagen production by microRNA-29b in cultured human stellate cells. Biochem. Biophys. Res. Commun. 391, 316–321 (2010) . 19913496 10.1016/j.bbrc.2009.11.056

[b25] Bartel D. P. MicroRNAs: target recognition and regulatory functions. Cell 136, 215–233 (2009) . 19167326 10.1016/j.cell.2009.01.002 PMC3794896

[b26] Ma F. *et al.* The microRNA miR-29 controls innate and adaptive immune responses to intracellular bacterial infection by targeting interferon-gamma. Nat. Immunol. 12, 861–869 (2011) . 21785411 10.1038/ni.2073

[b27] Grimson A. *et al.* MicroRNA targeting specificity in mammals: determinants beyond seed pairing. Mol. Cell 27, 91–105 (2007) . 17612493 10.1016/j.molcel.2007.06.017 PMC3800283

[b28] Maurer B. *et al.* MicroRNA-29, a key regulator of collagen expression in systemic sclerosis. Arthritis Rheum. 62, 1733–1743 (2010) . 20201077 10.1002/art.27443

[b29] Di Giammartino D. C., Nishida K. & Manley J. L. Mechanisms and consequences of alternative polyadenylation. Mol. Cell 43, 853–866 (2011) . 21925375 10.1016/j.molcel.2011.08.017 PMC3194005

[b30] Basu S., Binder R. J., Suto R., Anderson K. M. & Srivastava P. K. Necrotic but not apoptotic cell death releases heat shock proteins, which deliver a partial maturation signal to dendritic cells and activate the NF-kappa B pathway. Int. Immunol. 12, 1539–1546 (2000) . 11058573 10.1093/intimm/12.11.1539

[b31] Scaffidi P., Misteli T. & Bianchi M. E. Release of chromatin protein HMGB1 by necrotic cells triggers inflammation. Nature 418, 191–195 (2002) . 12110890 10.1038/nature00858

[b32] Shi Y., Evans J. E. & Rock K. L. Molecular identification of a danger signal that alerts the immune system to dying cells. Nature 425, 516–521 (2003) . 14520412 10.1038/nature01991

[b33] Chen C. J. *et al.* Identification of a key pathway required for the sterile inflammatory response triggered by dying cells. Nat. Med. 13, 851–856 (2007) . 17572686 10.1038/nm1603

[b34] Eigenbrod T., Park J. H., Harder J., Iwakura Y. & Nunez G. Cutting edge: critical role for mesothelial cells in necrosis-induced inflammation through the recognition of IL-1 alpha released from dying cells. J. Immunol. 181, 8194–8198 (2008) . 19050234 10.4049/jimmunol.181.12.8194 PMC2762646

[b35] Moussion C., Ortega N. & Girard J. P. The IL-1-like cytokine IL-33 is constitutively expressed in the nucleus of endothelial cells and epithelial cells in vivo: a novel 'alarmin'? PLoS ONE 3, e3331 (2008) . 18836528 10.1371/journal.pone.0003331 PMC2556082

[b36] Cayrol C. & Girard J. P. The IL-1-like cytokine IL-33 is inactivated after maturation by caspase-1. Proc. Natl Acad. Sci. USA 106, 9021–9026 (2009) . 19439663 10.1073/pnas.0812690106 PMC2690027

[b37] James R., Kesturu G., Balian G. & Chhabra A. B. Tendon: biology, biomechanics, repair, growth factors, and evolving treatment options. J. Hand Surg. Am. 33, 102–112 (2008) . 18261674 10.1016/j.jhsa.2007.09.007

[b38] Brown B. D. & Naldini L. Exploiting and antagonizing microRNA regulation for therapeutic and experimental applications. Nat. Rev. Genet. 10, 578–585 (2009) . 19609263 10.1038/nrg2628

[b39] Abonnenc M. *et al.* Extracellular matrix secretion by cardiac fibroblasts: role of microRNA-29b and microRNA-30c. Circ. Res. 113, 1138–1147 (2013) . 24006456 10.1161/CIRCRESAHA.113.302400

[b40] Roderburg C. *et al.* Micro-RNA profiling reveals a role for miR-29 in human and murine liver fibrosis. Hepatology 53, 209–218 (2011) . 20890893 10.1002/hep.23922

[b41] Sengupta S. *et al.* MicroRNA 29c is down-regulated in nasopharyngeal carcinomas, up-regulating mRNAs encoding extracellular matrix proteins. Proc. Natl Acad. Sci. USA 105, 5874–5878 (2008) . 18390668 10.1073/pnas.0801130105 PMC2311339

[b42] Abrahams Y., Laguette M. J., Prince S. & Collins M. Polymorphisms within the COL5A1 3'-UTR that alters mRNA structure and the MIR608 gene are associated with Achilles tendinopathy. Ann. Hum. Genet. 77, 204–214 (2013) . 23347277 10.1111/ahg.12013

[b43] Mendias C. L., Gumucio J. P. & Lynch E. B. Mechanical loading and TGF-beta change the expression of multiple miRNAs in tendon fibroblasts. J. Appl. Physiol. (1985) 113, 56–62 (2012) . 22539168 10.1152/japplphysiol.00301.2012 PMC3404830

[b44] Cushing L. *et al.* miR-29 is a major regulator of genes associated with pulmonary fibrosis. Am. J. Respir. Cell Mol. Biol. 45, 287–294 (2011) . 20971881 10.1165/rcmb.2010-0323OC PMC3175558

[b45] Wei W. *et al.* miR-29 targets Akt3 to reduce proliferation and facilitate differentiation of myoblasts in skeletal muscle development. Cell Death Dis. 4, e668 (2013) . 23764849 10.1038/cddis.2013.184 PMC3698551

[b46] Millar N. L. *et al.* Hypoxia: a critical regulator of early human tendinopathy. Ann. Rheum. Dis. 71, 302–310 (2012) . 21972243 10.1136/ard.2011.154229

[b47] Millar N. L., Wei A. Q., Molloy T. J., Bonar F. & Murrell G. A. Heat shock protein and apoptosis in supraspinatus tendinopathy. Clin. Orthop. Relat. Res. 466, 1569–1576 (2008) . 18459030 10.1007/s11999-008-0265-9 PMC2505259

[b48] Millar N. L., Wu X., Tantau R., Silverstone E. & Murrell G. A. Open versus two forms of arthroscopic rotator cuff repair. Clin. Orthop. Relat. Res. 467, 966–978 (2009) . 19184264 10.1007/s11999-009-0706-0 PMC2650068

[b49] McInnes I. B. *et al.* Production of nitric oxide in the synovial membrane of rheumatoid and osteoarthritis patients. J. Exp. Med. 184, 1519–1524 (1996) . 8879223 10.1084/jem.184.4.1519 PMC2192822

[b50] Dirks R. C. & Warden S. J. Models for the study of tendinopathy. J. Musculoskelet. Neuronal. Interact. 11, 141–149 (2011) . 21625051

[b51] Khan K. M., Cook J. L., Bonar F., Harcourt P. & Astrom M. Histopathology of common tendinopathies. Update and implications for clinical management. Sports Med. 27, 393–408 (1999) . 10418074 10.2165/00007256-199927060-00004

[b52] Kurowska-Stolarska M. *et al.* IL-33 induces antigen-specific IL-5+ T cells and promotes allergic-induced airway inflammation independent of IL-4. J. Immunol. 181, 4780–4790 (2008) . 18802081 10.4049/jimmunol.181.7.4780

[b53] Zheng Y. *et al.* Interleukin-22, a T(H)17 cytokine, mediates IL-23-induced dermal inflammation and acanthosis. Nature 445, 648–651 (2007) . 17187052 10.1038/nature05505

[b54] Hedrick M. N. *et al.* CCR6 is required for IL-23-induced psoriasis-like inflammation in mice. J. Clin. Invest. 119, 2317–2329 (2009) . 19662682 10.1172/JCI37378 PMC2719919

