# Supplementary material for: MicroRNA29a regulates IL-33-mediated tissue remodelling in tendon disease
Source: Nat Commun. 2015 Apr 10;6:6774. doi: 10.1038/ncomms7774 (PMC4403384; doi:10.1038/ncomms7774)
Supplement: Supplementary Information — Supplementary Figures 1-7, Supplementary Table 1 [file ncomms7774-s1.pdf]

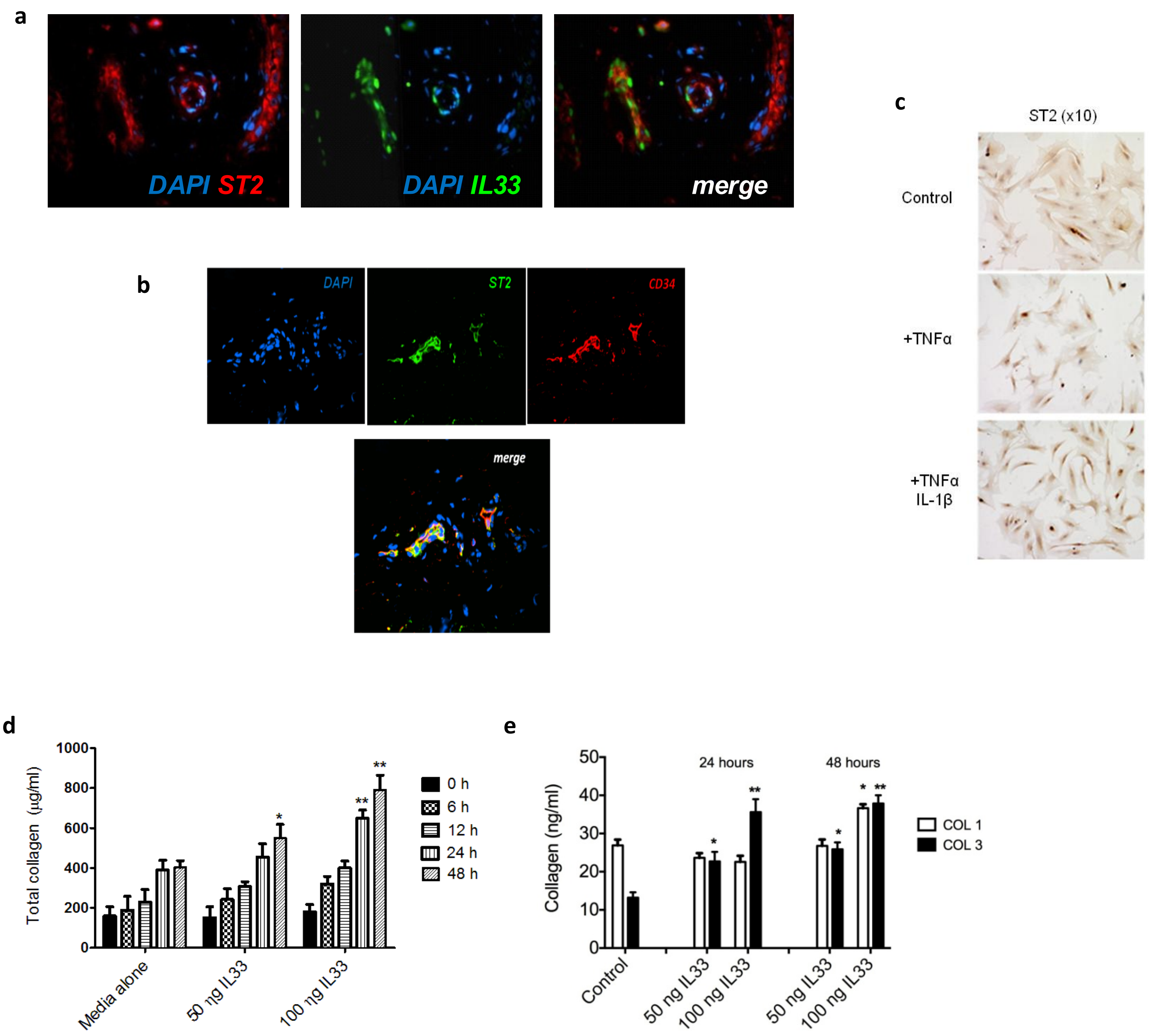

Supplementary Figure 1

Supplementary Figure 1

Expression of IL-33, ST2 and the production of collagens and cytokines by human tenocytes. (**a**) Double immunofluorescence staining of IL33 and ST2 in perivascular regions and tenocytes in human tendinopathy ('early pathology'). (**b**) ST2 and CD34 double positive cells staining in vascular region. (**c**) ST2 staining in explanted control human tenocytes cultured with medium alone, 100 ng/ml TNFα, or 100 ng/ml TNFα + 100 ng/ml IL-1β. (**d**) Time course of total collagen production in the supernatant by human control tenocytes cultured with rhIL-33. (**e**) Collagen 1 and 3 concentrations in the supernatant of control human tenocytes cultured with rIL-33. Data in **d, e** are mean ± SD of triplicate samples determined by ELISA, representative of three individual patient samples. *$p<0.05$, **$p<0.01$ vs corresponding control samples. (Mann-Whitney).

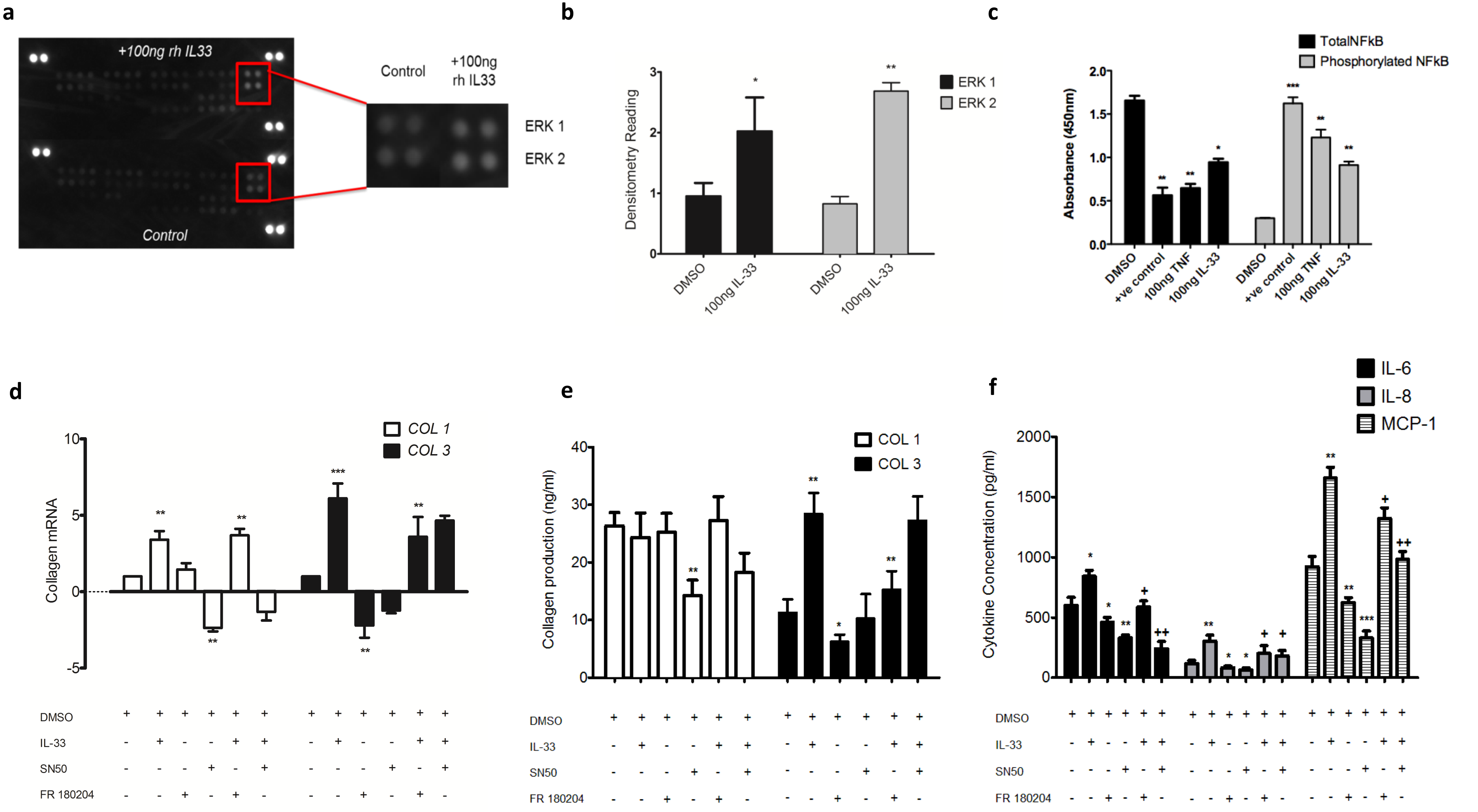

Supplementary Figure 2

Supplementary Figure 2

The role of ERK and NFκB in the production of collagen and cytokines by IL-33-activated human tenocytes. (**a**) Whole-cell lysates of control human tendon explant were examined by Human Phospho-MAPK Array for the phosphorylation of MAPK's 1 h after cultured with rhIL-33. (**b**) The fold change of MAPK's was determined by densitometry and normalised to the control sample on the array. Data are mean fold change ± SD of duplicate samples, representative of five individual donors of tendon explant tissue. *p<0.05, **p<0.01 compared to DMSO control (Student's *t*-test). (**c**) Whole-cell lysates were examined by ELISA for total NFκB and phosphorylated NFκB in tenocyte cultured for 24 h with TNFα and IL-33. Data are mean ± SD of three individual experiments. *p<0.05, **p<0.01, ***p<0.001 vs respective DMSO controls. (Student's *t*-test). (**d-f**) Control human tenocytes were cultured as indicated for 24 h with rhIL-33 and ERK (FR 180204) or NFκB (SN050) inhibitor. (**d**) Levels of mRNA for Collagen 1 and 3 in the cells were determined by q-PCR. (**e**) Concentrations of Collagen 1 and 3 protein (ELISA) and (**f**) concentrations of cytokine (Luminex) in the supernatant were determined. Data are mean ± SD of triplicate samples and represent experiments on five individual patient samples. *p<0.05, **p<0.01, ***p<0.01 vs control samples (DMSO). $^{+}$p<0.05, $^{++}$p<0.01 vs DMSO+IL-33 (ANOVA).

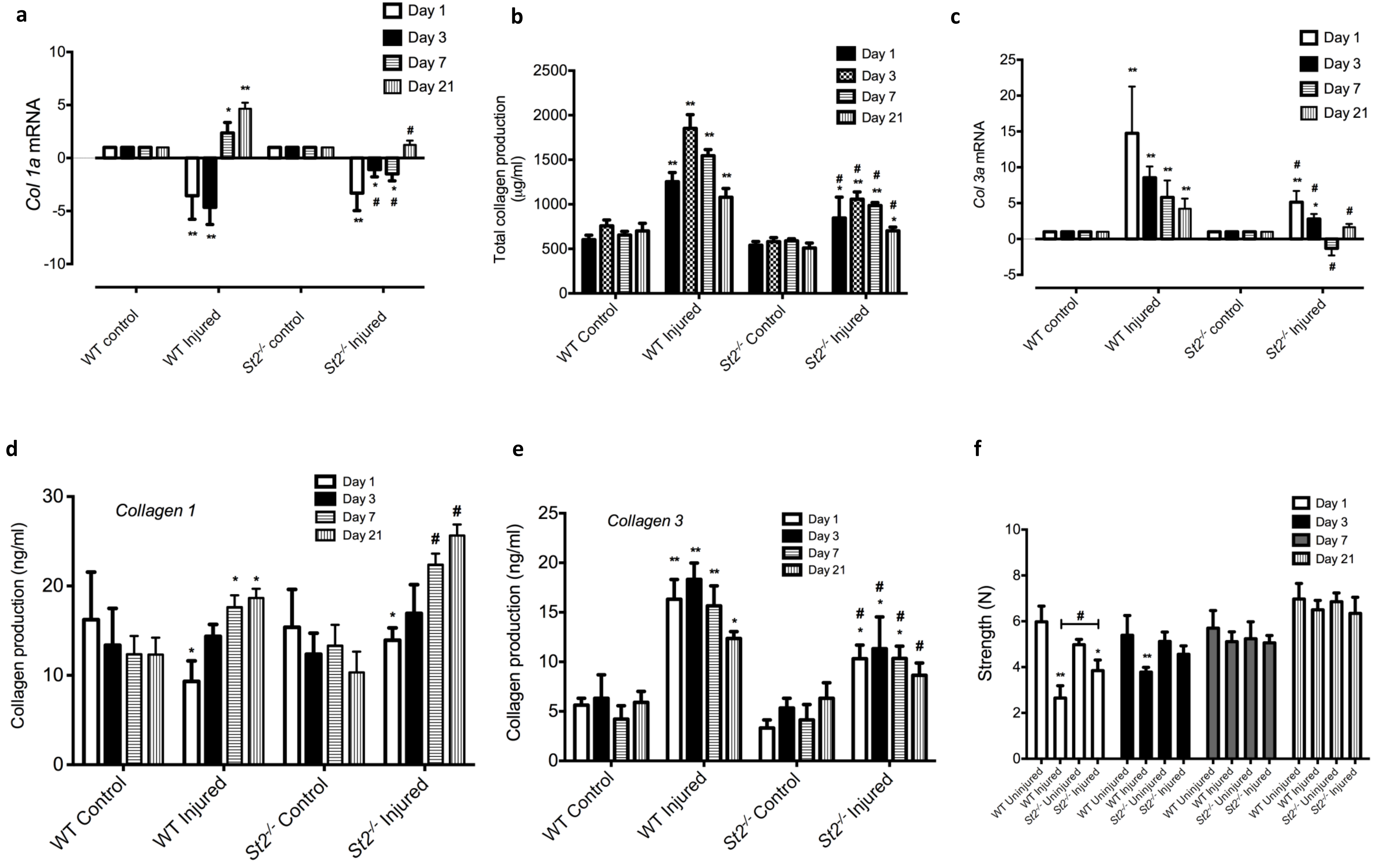

Supplementary Figure 3

Supplementary Figure 3

Effect of ST2-deficiency on injury-induced collagen production by the tendon and tendon strength. (**a**) *Col1a* message levels in the tendon of WT and *St2*$^{-/-}$ mice post injury determined by q-PCR. *p<0.05, **p<0.01 WT/*St2-/-* injured mice vs control; # p<0.05, *St2*$^{-/-}$ injured mice vs WT injured mice. (ANOVA). (**b**) Total collagen level in the tendon of WT and *St2*$^{-/-}$ mice post injury determined by Sircol assay. *p<0.05, **p<0.01 WT/ *St2*$^{-/-}$ injured mice vs control; # p<0.01, *St2*$^{-/-}$ injured mice vs WT injured mice. (ANOVA). (**c**) *Col3a* message levels in the tendon of WT and *St2*$^{-/-}$ mice post injury determined by q-PCR. Data are mean fold change ± SD of duplicate samples, representative of four mice per group. *p<0.05, **p<0.01 WT/ *St2*$^{-/-}$ injured mice vs control; # p<0.01, *St2*$^{-/-}$ injured mice vs WT injured mice. (ANOVA). (**d,e**) Collagen 1 and 3 protein levels post injury was determined by ELISA. WT mice showed significantly greater production of Collagen 1 on days 7 and 21 post injury compared to *St2*$^{-/-}$ mice in the injured groups. The most significant difference is the lack of collagen 3 production in *St2*$^{-/-}$ mice at all time pints post injury compared to the injured WT mice group. Data are mean fold change ± SD of duplicate samples representative of four mice per group. *p<0.05, **p<0.01 WT/*St2*$^{-/-}$ injured mice vs control; # p<0.01, *St2*$^{-/-}$ injured mice vs WT injured mice. (ANOVA). (**f**) Time course of tensile strength (load to failure, N=Newton's (force/unit area)) for WT and *St2*$^{-/-}$ mice injured and uninjured tendons. Data are mean fold change ± SD of duplicate samples, representative of four mice per group. *p<0.05, **p<0.01 control vs injured mice. $^{+}$p<0.05, $^{++}$p<0.01, WT injured vs *St2*$^{-/-}$ injured mice. (ANOVA). Data for mRNA are relative to *18s* housekeeping gene.

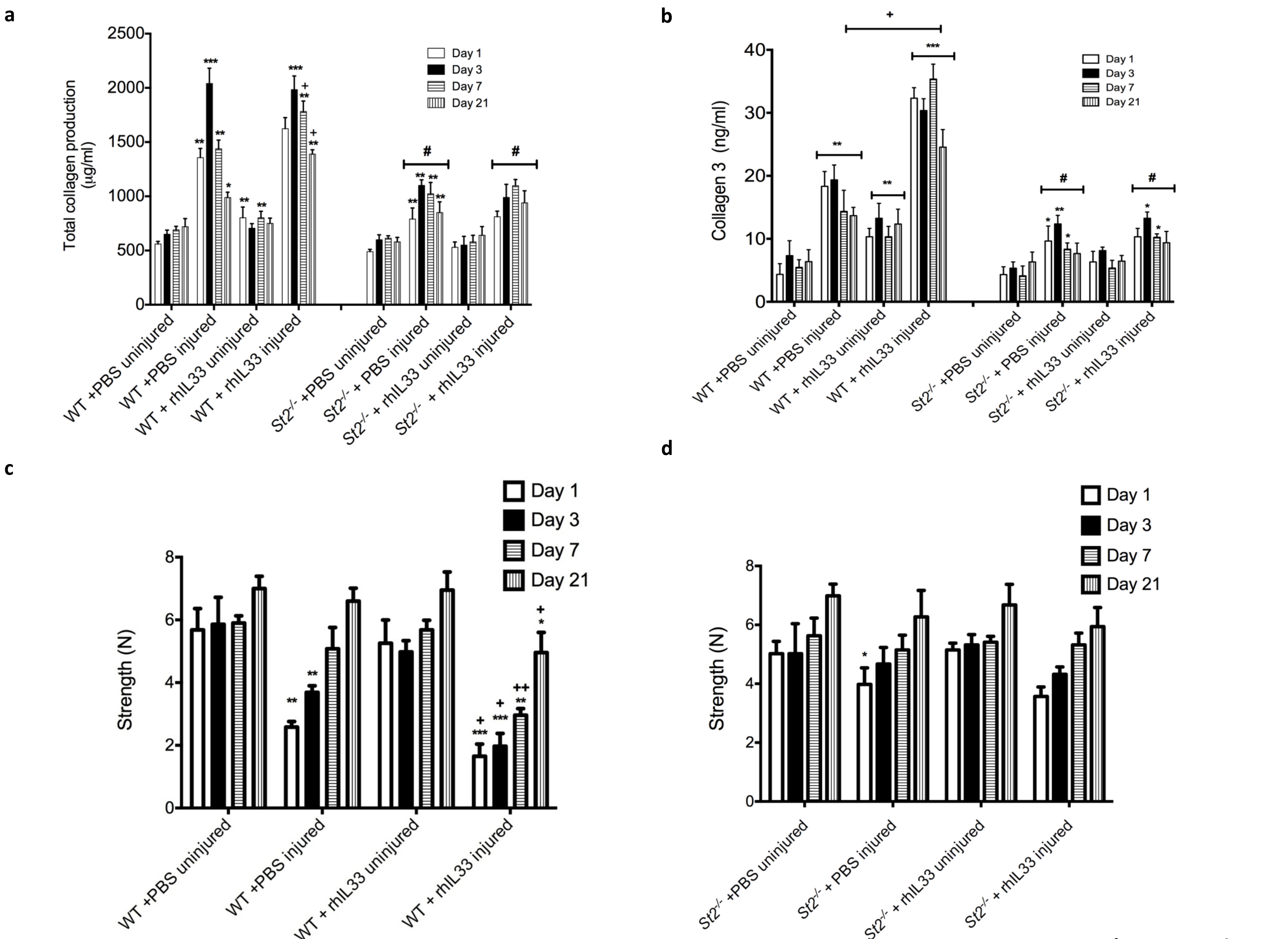

Supplementary Figure 4

Supplementary Figure 4

Effect of IL-33 on collagen production and tensile strength of murine tendon. (a) Total collagen production (Sircol) and (b) Collagen 3 protein (ELISA) in WT and St2-/- mice treated with rhIL-33. The most significant shift was in the production of Collagen 3 protein in WT mice treated with rhIL-33 at all-time points post injury. However significant differences were only noted between PBS injured mice vs rhIL-33 injured mice on Days 7 and 21, suggesting that endogenous IL-33 is likely responsible for the majority of early collagen changes. Data are mean fold change ± SD of duplicate samples and are representative of 4 mice per group. *$p<0.05$,**$p<0.01$, ***$p<0.001$ injured mice vs uninjured mice. # $p<0.05$, WT vs St2-/- mice. (ANOVA). (c, d) Tendon strength of WT and St2-/- mice treated with IL-33. rhIL-33 treatment led to a significantly decreased in tensile strength in WT uninjured and injured mice (c) but not in St2-/- mice (d) on Days 1 and 3 of the treatment. WT injured mice had 45% decrease in strength when treated with rhIL-33 on days 1 and 3 post injury, and with 20% decrease in strength on 21 says post injury compared to PBS injection alone. Data are mean fold change ± SD of duplicate samples, representative of 4 mice per group. *$p<0.05$,**$p<0.01$, ***$p<0.001$ injured vs uninjured mice. + $p<0.05$, ++ $p<0.01$, WT vs St2-/- mice. (ANOVA).

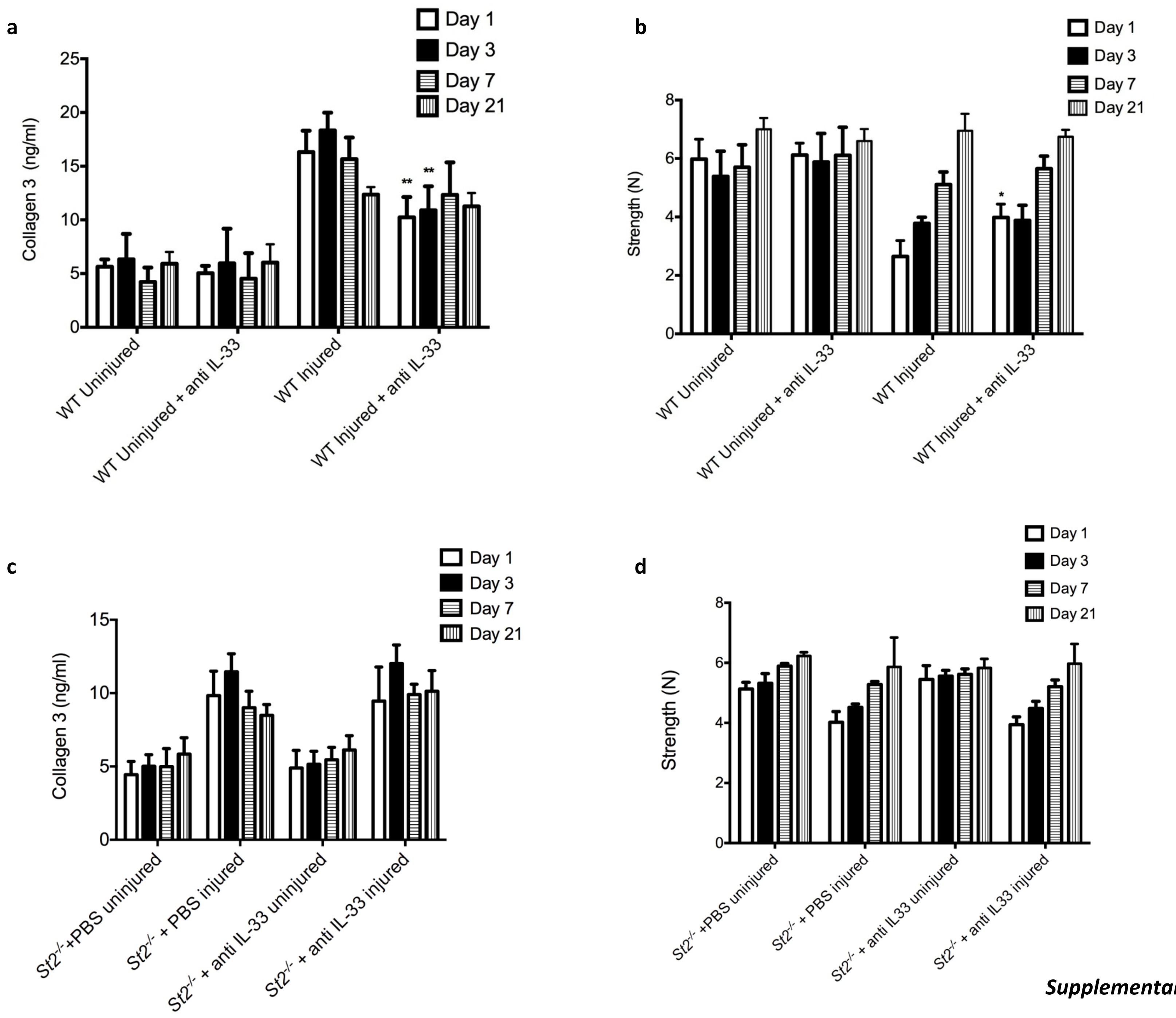

Supplementary Figure 5

Supplementary Figure 5

Collagen 3 production and tensile strength of tendon in mice treated with anti-IL-33 antibody. (**a, c**) Time course of collagen 3 protein production (ELISA) and (**b, d**) tensile strength in WT (**a**, **b**) and *St2*$^{-/-}$ (**c**, **d**) injured or uninjured mice treated with anti-IL33 antibody or not. Data are mean fold change ± SD of duplicate samples, representative of 4 mice per group. *p<0.05, **p<0.01, WT + anti-IL33 vs WT injured (ANOVA).

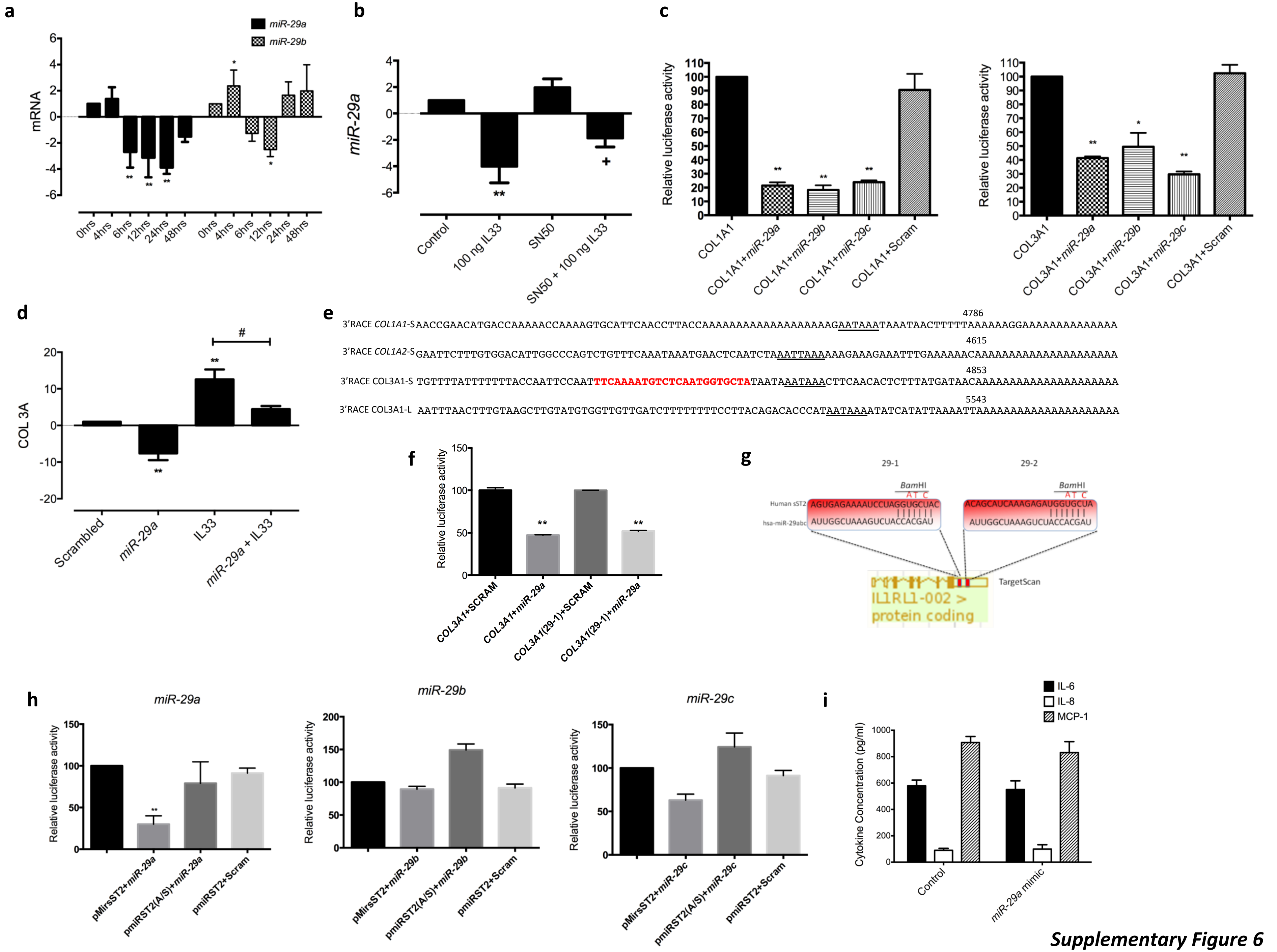

Supplementary Figure 6

Supplementary Figure 6

miR29a biology. (**a**) Time course of *miR-29a* and *miR-29b* expression in human tenocytes (normal controls) cultured with IL-33 *in vitro*. *p<0.05, **p<0.01 vs 0 h. (Student's *t* test). (**b**) Control human tenocytes were cultured as indicated for 24 h with rhIL-33 and /or NFκB (SN050) inhibitor. **p<0.01 vs DMSO control. $^{+}$ p<0.05 vs 100 ng IL-33 (Student's *t*-test). Levels of *miR-29* in the cells were determined by q-PCR relative to *U6* housekeeping gene. Data are mean ± SD of triplicate cultures, representative of 3 independent experiments. (**c**) Luciferase activity in HEK 293 cells transfected with precursor *miR-29 a/b/c* (pre-miR-29) containing 3′UTR of *COL1* or *COL3*. Activity was determined relative to controls transfected with scrambled RNA (defined as 100%). Data are mean ± SD of triplicate cultures, representative of 3 independent experiments. *p<0.05, **p<0.01 vs scrambled control. (Mann Whitney). (**d**) *COL3* gene expression following addition of *miR29a* mimic and/or 100 ng rhIL-33 in human tenocytes relative to *GAPDH* housekeeping gene. Data are mean ± SD (n=3). **p<0.01 vs scrambled control, $^{\#}$p<0.05.. (Student-*t* test). (**e**) Sequences of 3′RACE products of tenocyte collagen transcripts. Polyadenylation signals are underlined *miR-29a* binding site shown in red. (**f**) Luciferase assay on *COL3A1* 29a MRE site mutated luciferase reporter construct. Activity was determined relative to controls transfected with scrambled RNA (defined as 100%). Data are mean ± SD of triplicate cultures, representative of 3 independent experiments. **p<0.01 vs scrambled control. (Mann Whitney). (**g**) Figure showing seed regions of the two Targetscan predicted *miR-29a* MRE sites: 29-1 and 29-2. (**h**) Co-transfection of HEK 293 cells with pre-miR29a, b. c containing 3′UTR of soluble *ST2*. A/S, antisense. *miR-29a* significantly reduced the relative luciferase activity compared with scrambled RNA–transfected controls (gene + scram indicates positive control), (n = 3). **p<0.01 vs control. (Mann Whitney). (**i**) IL-6, IL-8 and MCP-1 production in tenocytes cultured with *miR-29a* mimic and analyzed by Luminex. Data are mean ± SD of duplicate samples, representative of five individual experiments (Mann-Whitney).

Please indicate if the tenocytes were from the "control" group, for all cases human tenocytes are used.

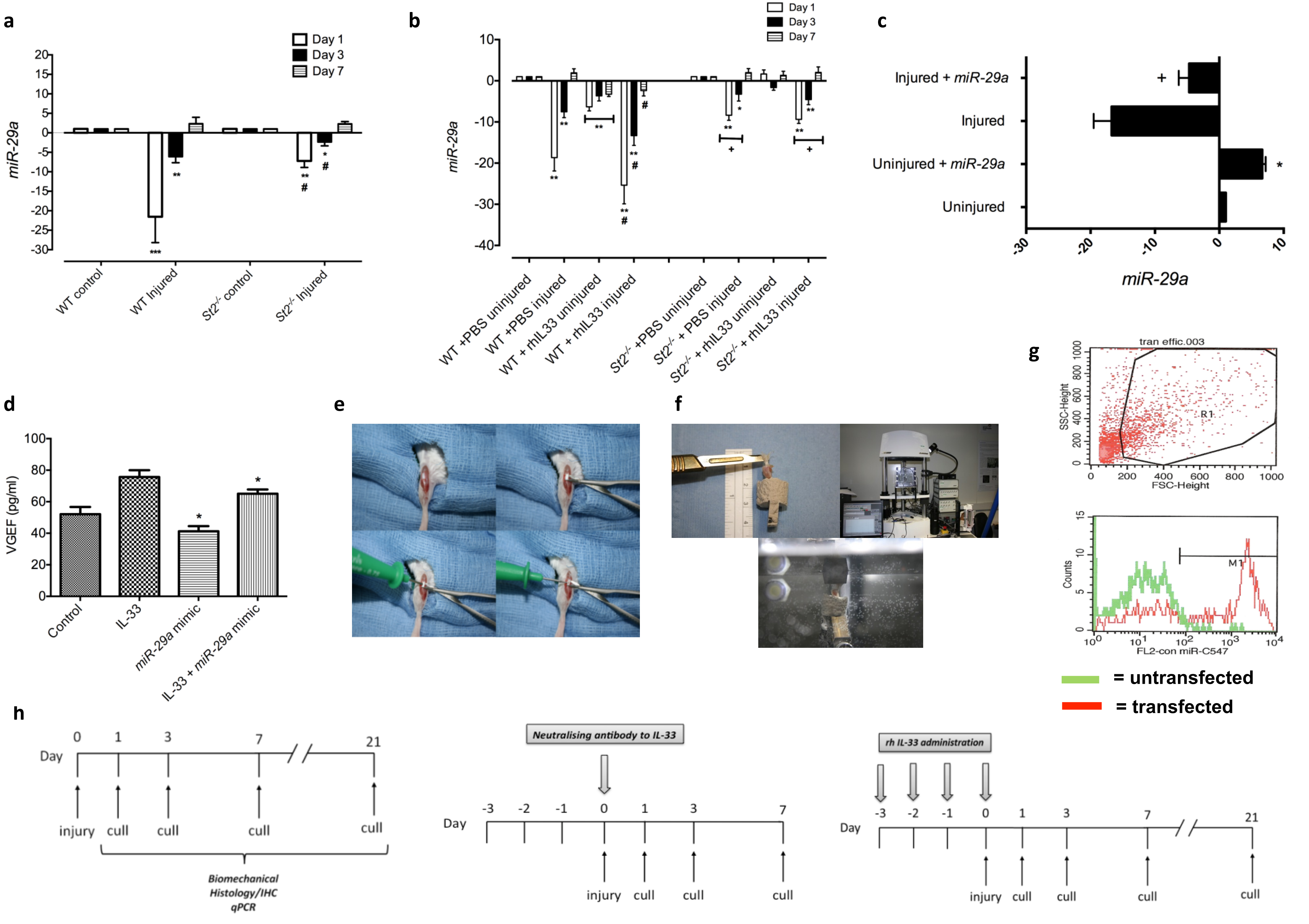

Supplementary Figure 7

Supplementary Figure 7

miR-29a expression *in vivo* and experimental protocols**.** (**a**) Quantitative PCR showing time course of *miR-29a* expression in the tendon of WT and *St2*$^{-/-}$ mice. Data are mean fold change ± SD of duplicate samples, representative of 4 mice per group. *p<0.05,**p<0.01, ***p<0.001, injured versus uninjured mice. $^{\#}$p<0.05, WT versus *St2*$^{-/-}$ mice. [ANOVA]. (**b**) Quantitative PCR showing mean fold change ± SD in *miR-29a* in WT and *St2*$^{-/-}$ mice in injured versus uninjured animals following treatment with rhIL-33 or PBS. *p<0.05,**p<0.01, ***p<0.001, injured versus uninjured mice. $^{+}$p<0.05, WT versus *St2*$^{-/-}$ mice. (ANOVA) (**c**) Quantitative PCR showing fold change (mean ± SD) in *miR-29a* in WT mice in injured versus uninjured animals following treatment with mir29a or PBS on day 1 post injury. *p<0.05 uninjured PBS vs uninjured +miR-29a mimic, $^{+}$p<0.01 injured PBS vs injured + *miR-29a* (ANOVA). Levels of *miR-29a* are relative to *U6* housekeeping gene. (**d**) VGEF protein expression in tenocytes treated with 100 ng rhIL-33 and *miR-29a* mimic. (n=3), *p<0.05 vs untreated control. (Student-*t* test). (**e**) Photographs showing the patellar injury model. From top left clockwise; the tendon is exposed through a midline incision; scissors are passed behind the tendon to divide the overlying retinaculum and a 0.75 mm punch is placed through the mid-substance of the tendon. (**f**) Photograph of harvested tibia and attached patellar tendon and patella set in Isopon fixative prior to testing. The BOSE ElectroForce® 3200 test instrument used to test biomechanical strength. (**g**) Transfection efficiency was assessed by flow cytometry using labelled D547 mimic in human tenocytes. Transfection rates was >75%. (**h**) Protocols for *in vivo* experiment and treatment with rhIL-33 /anti IL-33.

Tear Size	Control	Small (<1cm^2)	Medium(>1-3cm^2)	Large (>3-5cm^2)	Massive (>5cm^2)
Number of cases	10	6	6	3	2
Male:Female ratio	6:4	4:2	4:2	2:1	1:1
Mean age in years(range)	30 (18-38)	50 (39-58)	54 (50-62)	54 (47-60)	63 (50-71)
Mean duration of symptoms in months (range)	8.7 (1-20)	5.9 (2-15)	7.2 (3-19)	8.5 (4-18)	5.7 (2-12)
Mean number of steroid injections	0	2.0	1.6	1.2	1.3

Supplementary Table 1

Patient details and demographics
